# Evaluation of Metaplastic Triple-Negative Breast Cancer Extracellular Matrix Structure and Protein Composition

**DOI:** 10.3390/bioengineering13010047

**Published:** 2025-12-31

**Authors:** Jonathan J. Savoie, Katherine L. Hebert, Connor T. King, Emily C. McConnell, Van T. Hoang, W. Todd Monroe, Matthew E. Burow, Bridgette M. Collins-Burow, Jorge A. Belgodere, Elizabeth C. Martin

**Affiliations:** 1 Department of Biological and Agricultural Engineering, Louisiana State University, Baton Rouge, LA 70803, USAtmonroe@lsu.edu (W.T.M.); 2 Section of Hematology & Medical Oncology, Department of Medicine, Tulane University School of Medicine, New Orleans, LA 70112, USA; 3 Tulane Cancer Center, Tulane University School of Medicine, New Orleans, LA 70112, USA

**Keywords:** metaplastic breast cancer, extracellular matrix, MFAP2

## Abstract

Alterations in the tumor extracellular environment and matrix stiffness promote tumor progression. Furthermore, correlational studies have identified enrichment of extracellular matrix (ECM) proteins (glycoproteins, collagens) in breast tumors. Despite these findings, there has yet to be an interdisciplinary analysis of both ECM composition and structural architecture in rare breast tumors, such as metaplastic breast cancer. Here, we explored changes in ECM protein expression and architecture in a triple-negative breast cancer (TNBC) metaplastic tumor through SEM, proteomics, and RNA sequencing. SEM revealed that the tumor pore size was larger compared to the control adipose tissue. Oscillating rheometry demonstrated increased ECM stiffness in the tumor compared to the control adipose breast adipose. Proteomic analysis of the metaplastic TNBC tumor showed significant enrichment for ECM proteins, notably glycoproteins compared to the control adipose. Interestingly, these samples showed no observed changes in expression for major fibrillar collagens COL1A1 and COL1A2, and a reduced expression of COL3A1. To determine the impact of less characterized ECMs in metaplastic TNBC, we overexpressed MFAP2 in primary metaplastic breast cancer cells and performed RNA sequencing. MFAP2 overexpression was associated with upregulation of epithelial-to-mesenchymal transition-related genes. Overall, our results establish an extracellular signature and onco-architecture for the metaplastic triple-negative tumor type.

## 1. Introduction

Our comprehension of breast cancer progression has increased dramatically over the past decade since the discovery that the cell extrinsic stromal microenvironment can drive tumorigenesis in a comparable manner to oncogenic mutations and cell intrinsic modifications [1]. The extracellular matrix (ECM) has garnered increased attention in recent years and research has focused on the ECM in promoting cancer progression [2]. Consisting of a complex network of cross-linked structural and regulatory proteins, ECM proteins are comprised of collagens, glycoproteins, proteoglycans, and matrix-associated proteins with distinct biological roles in maintaining normal cellular processes, including regulation of tissue development and tissue homeostasis. At the onset of tumor development, the ECM undergoes a dynamic change in protein composition where ECM components are deposited, degraded, or otherwise modified [3]. Consequently, changes in biomechanical and biochemical properties deregulate cellular function and promote a malignant cellular response [4]. Moreover, it is suggested that ECM composition signatures are breast cancer subtype specific [5]. Proteomic analyses of human breast tumors compared with adjacent breast tissue showed differences in breast cancer ECM protein composition among breast cancer subtypes [6]. In accordance with this, total collagen content and collagen I expression were elevated in hormone receptor-positive tumors compared to TNBC [5,7], and low expression of collagen I was associated with more aggressive cancers [7]. Elevated collagen and fiber deposition in aggressive tumors, such as TNBC, have been reported by others; however, these instances focused on comparisons within TNBC such as race-driven differences [8] or therapeutic response [7,9]. These findings highlight the variability of ECMs across tumor types and the need for increased characterization. Finally, distinct ECM compositions can induce different physical properties, thereby affecting cellular behavior across the breast cancer subtypes [4,5].

In addition to alterations in protein expression, remodeling of ECM architectural structure can also occur through modifications in matrix stiffness, fiber alignment, and porosity. This is critical when considering that matrix structure is responsible for providing structural support, tissue maintenance, directing tissue remodeling, application of mechanical forces, and diffusion of growth factors, nutrients, and other biochemical molecules [10]. Disordered ECM architecture associated with cancer development can disrupt the delicate balance of applied mechanical forces exerted by the matrix onto cells, distorting cell shape and altering gene expression and impacting cellular processes [11]. Changes in ECM architecture interference with cell–matrix interactions have been shown to not only stimulate mammary cell proliferation and survival pathways but also re-program the surrounding stromal cells [12]. Furthermore, ECM remodeling can induce epithelial-to-mesenchymal transition (EMT) and promote metastasis, driving cancer progression to advanced stages [13]. Consequently, the changes that occur in both structural features and protein composition can significantly dysregulate normal tissue function and create a microenvironment favorable to cancer development [14].

Despite research efforts dedicated to elucidating the role of ECM modification in breast cancer [15], to our knowledge, there has yet to be an interdisciplinary approach that investigates both changes in matrix composition and structural alterations in ECM such as alignment and porosity in rare breast cancers. Increased understanding of the cell extrinsic profile of rare tumor types will enhance our ability to construct accurate 3D tumor models for these rare malignancies. Metaplastic breast cancer represents a rare, highly aggressive type of breast cancer occurring in less than 1–5% of breast cancer patients [16,17]. It is characterized by poor survival and typically presents as receptor-negative [16,17]. Recent studies suggest remodeling of the ECM composition in metaplastic breast cancer compared to non-metaplastic TNBC [15]; however, the metaplastic ECM has not yet been evaluated in a cell-free system. Here, we perform a pilot study and aim to elucidate alterations in ECM protein expression and fiber characteristics in a rare metaplastic triple-negative tumor. As this is a rare tumor type, there were limited fresh biospecimens for decellularization and results are representative of a single tumor. We compared ECM properties in a single decellularized metaplastic triple-negative tumor sample and adjacent breast tissue (tumor-adjacent), and compared these biospecimens to distant, non-cancerous breast tissue biopsied from the opposite side of the same breast (control adipose). ECM protein expression was determined by proteomics, and ECM porosity, fiber thickness, and fiber alignment were assessed by scanning electron imaging (SEM). In addition, ECM stiffness between tumor-adjacent and control adipose breast tissue was determined through oscillating rheometry readings. Collectively, these analyses characterize ECM features in single metastatic TNBC tumors and provide a preliminary platform for future disease modeling of this rare disease.

## 2. Materials and Methods

### 2.1. Tissue Collection

Tissue samples were acquired in collaboration with the Louisiana Cancer Research Consortium Biospecimen Core. Samples collected include primary tumor tissue, breast tissue biopsied adjacent to tumor site (tumor-adjacent), and distant, normal breast tissue biopsied from the opposite side of the same breast (control adipose). Control adipose tissue was used as a baseline expression to gauge measured fold differences in both tumor and tumor-adjacent samples. Tumor tissue from donor TU-BcX-4IC (4IC) is a rare, metaplastic breast carcinoma taken from sub-areolar and lateral areas, which had invaded into the pectoralis major muscle. It is characterized by rapid growth, high rates of metastasis and recurrence, and drug resistance [18]. In addition, tissue samples were taken from a supplementary triple-negative donor TU-BcX-4M4 (4M4) [19] and tested to assess any commonalities between metaplastic (4IC) and TNBC (4M4) tumor-adjacent tissue.

### 2.2. Cell Culture

The metaplastic TNBC cell line TU-BcX-4IC, previously established from our lab [18], and non-metaplastic TNBC cell line MDA-MB-231 (ATCC, Manassas, VA, USA) were cultured in Dulbecco’s Modified Eagle Medium (DMEM) (Gibco, Waltham, MA, USA) supplemented with 10% HyClone Cosmic Calf Serum (Cytiva, Wilmington, DE, USA), 1% minimal essential amino acids (Gibco, Waltham, MA, USA), 1% non-essential amino acids (Gibco, Waltham, MA, USA), 1% antibiotic/anti-mycotic (Gibco, Waltham, MA, USA), and insulin (Gibco, Waltham, MA, USA) under mycoplasma-free conditions at 37 °C and 5% *v*/*v* CO_2_.

### 2.3. MFAP2 over Expression (OE) Cell Lines

MDA-MB-231 and TU-BcX-4IC cells were cultured in 10 cm^2^ dishes (VWR, Inc., Radnor, PA, USA) at 100,000 cells/mL in complete media. After 24 h, cells were transfected with 5 μg of pCMV6-Entry Mammalian Expression Vector DNA (Origene, Rockville, MA, USA) and MAGP1 5 µg (MFAP2) (NM_017459) Human Tagged ORF Clone (Origene, Rockville, MA, USA). The DNA clones were mixed with 15 μL of TF8 1001 transfection reagent (Origene, Rockville, MA, USA) (1:3 ratio of DNA to reagent) in 4 mL of DMEM supplemented with 10% Fetal Bovine Serum (FBS; Hyclone, Logan, UT, USA), 1% nonessential amino acids, minimal amino acids, sodium pyruvate, antibiotic/anti-myotic, and insulin 15 min prior to adding the transfection complexes drop-wise onto the cells. After 48 h, media was aspirated and replaced with media for selection, containing geneticin (Thermo Fisher, Scientific, Waltham, MA, USA) at 200 μg/mL. Cells were grown to 80% confluency and MFAP2 overexpression was confirmed through qRT-PCR, as limited available antibodies exist for this ECM protein.

### 2.4. RNA Sequencing

Metaplastic TU-BcX-4IC and TNBC MDA-MB-231 cell lines with pCMV6 Vector or MFAP2 cells were cultured in a T75 flask (Thermo Scientific, Waltham, MA, USA) grown to 80% confluency and collected for RNA extraction. Total RNA was extracted from the cell lines using Quick RNA Miniprep Kit from Zymo Research (Irvine, CA, USA) and sent to Azenta (TU-BcX-4IC) (Burlington, MA, USA) or LSU Genomics Core Services (MDA-MB-231) (Baton Rouge, LA, USA) for RNA sequencing. The RNA sequencing workflow after RNA isolation included initial PolyA selection-based mRNA enrichment, mRNA fragmentation, and random priming with subsequent first- and second-strand complementary DMA (cDNA) synthesis. Afterward, end-repair 5’ phosphorylation and nucleotide (dA)-tailing were performed. Last, adaptor ligation, polymerase chain reaction (PCR), and Illumina NovaSeq technology-based sequencing with two 150-base pair (bp) read length were carried out. Reads were aligned to GSEA against nine different molecular signature databases. The statistical results were generated using DESeq2 (Version 1.48.1). Log2FC was used to estimate changes in gene expression, representing the ration of the gene expression between the two conditions. Aligned data files are available in Supplemental File S2 and raw fastq files are available upon request. Analysis between TU-BcX-4IC and MDA-MB-231 cell lines with Vector or MFAP2 OE was performed as follows: data sets for comparison where total gene changes were between pCMV6 MFAP2 OE vs. pCMV6 Vector. Fold changes were identified as increased if the gene log2 fold change compared to the parental sample was a positive value, and fold changes were identified as decreased if there was a negative value. All genes were considered significant if the adjusted *p*-value was *p* < 0.05. Identification of most significantly altered pathways was performed through Enrichr gene set analysis tool [20,21], specifically from the Hallmarks database (access date October 2025).

### 2.5. 2D Crystal Violet Viability Assay

TU-BcX-4IC pCMV6 Vector and pCMV6 MFAP2 OE cells were cultured in 96-well plates (VWR, Inc., PA, USA), 2000 cells per well in complete media, under mycoplasma-free and normal cell culture conditions. The following day (Day 0), cells were treated with docetaxel (0.1 nM, 1 nM, 10 nM, 100 nM), paclitaxel (0.1 nM, 1 nM, 10 nM, 100 nM), carboplatin (0.1 µM, 1 µM, 10 µM, 100 µM), or vehicle (DMSO/water). Plates were harvested on Day 3, fixed with 25% glutaraldehyde (Fisher Scientific), and stained with 1% crystal violet (Sigma-Aldrich, St. Louis, MO, USA) dissolved in a 10% methanol solution (*v*/*v* in water). Cells were lysed in 33% acetic acid (Sigma-Aldrich), and the absorbance was read at 590 nm on a plate reader (Cytation 5, Gen 5 version 3.12). Studies were performed in biological triplicates for each cell line. Changes in average cell density were determined by comparison to vehicle control. All compounds were purchased from Selleck Chemicals (Houston, TX, USA).

### 2.6. 3D Spheroid Viability Assay

To determine differences in chemotherapy response, 3D spheroids were generated with 5000 TU-BcX-4IC pCMV6 Vector or TU-BcX-4IC pCMV6 MFAP2 OE cells seeded in a 96-U-well bottom plate (Thermo Scientific, Waltham, MA, USA). After 24 h, cells were treated with docetaxel (0.1 nM, 1 nM, 10 nM, 100 nM), paclitaxel (0.1 nM, 1 nM, 10 nM, 100 nM), carboplatin (0.1 µM, 1 µM, 10 µM, 100 µM), or vehicle (DMSO/water) for 3 days. Spheroids were stained with LIVE/DEAD Viability/Cytotoxicity Kit for mammalian cells (Invitrogen, Waltham, MA, USA), according to the manufacture’s protocol. Images of the spheroids were taken and GFP mean intensity (live cells) and RFP mean intensity (dead cells) were analyzed on the Cytation 5 software (version 3.12). Studies were performed in triplicate for each cell line in independent experiments.

### 2.7. Tissue and Tumor Decellularization

Tissue samples were decellularized using modifications of previously established methods [22]. The samples were thawed, biopsy punched, and placed in PBS overnight at 4 °C. After, the samples were washed with deionized water and incubated with agitation in 0.1% Triton X-100 solution (Bio-Rad, Hercules, CA, USA). Next, the samples were washed in deionized water and incubated with agitation in 2% sodium deoxycholate solution, followed by additional washing in deionized water and incubated in saline with agitation (ThermoFisher Scientific, Waltham, MA, USA). Finally, the tissue was washed with deionized water treated with DNAse I (1 U/mL, Sigma-Aldrich, St. Louis, MO, USA) and stored in 5% antibiotic-antimycotic (100 U/mL; ThermoFisher Scientific, Waltham, MA, USA) in PBS at 4 °C until use.

### 2.8. Proteomics Analysis

Decellularized TU-BcX-4IC tumor, tumor-adjacent, and control adipose tissue samples were sent to Xavier University Proteomics Core Facility (New Orleans, LA, USA) for proteomic evaluation according to previously described methods [23]. In brief, decellularized tumor and adipose scaffolds were processed through denaturation, reduction, alkylation, and deglycosylation, followed by enzymatic digestion with LysC and trypsin. Acidification was then performed to halt enzymatic digestion, and samples were centrifuged, desalted, and analyzed with UV-vis spectroscopy at 280 nm. Peptides were then labeled with TMT reagents and analyzed by LC-MS/MS using an LTQ-Orbitrap XL system (ThermoFisher Scientific, Waltham, MA, USA), with resulting data provided in Supplemental File S1.

### 2.9. Database Search and TMT Quantification

The protein search algorithm used was Mascot v2.3.01 (Matrix Science, Boston, MA, USA). Mascot format files were generated by the Proteome Discoverer 1.2 software (Thermo-Fisher Scientific) using the following criteria: database, IPI_Human.fasta.v3.77 (containing 89,422 entries and concatenated with the reversed versions of all sequences.); enzyme, trypsin; maximum missed cleavages, 2; static modifications, carbamidomethylation (+57 Da), N-terminal TMT6plex (+229 Da), lysyl TMT6plex (+229 Da). Dynamic modifications, N-terminal Clnpyro- Glu (+17 Da); methionine oxidation (+16 Da); STY phosphorylation (+80 Da); precursor mass tolerance was set at 20 ppm; fragment match tolerance was set at 0.8 Da. Peptides reported by the search engine were accepted only if they met the false discovery rate of *p* < 0.05 (target decoy database), and a Mascot ion score ≥ 30 for peptide identifications was required. For TMT quantification, the ratios of TMT reporter ion abundances in MS/MS spectra generated by HCD (up to six reporter ions ranging from m/z 126.12 to m/z 131.14) from raw data sets were used to calculate fold changes in proteins between control adipose breast tissue and tumor-adjacent breast tissue or tumors.

### 2.10. Survival Correlational Analysis

Kaplan–Meier plots were created for both protein expression to assess overall survival and distant metastasis-free survival in breast cancer patients [24]. Plots were generated based on protein expression from 126 patients using the following criteria: database, Liu_2014; survival, split patients by auto selecting the best cutoff; follow up threshold for all patients; censor at threshold and compute median survival boxes; restrict analysis to ‘Negative’ for ER, PGR, and HER2 status; due to limited sample size, plotting was not restricted to any cohort, stage, grade, or lymph node status.

### 2.11. Scanning Electron Microscopy

Scanning electron imaging was performed on decellularized metaplastic tumor (4IC), metaplastic TNBC tumor tumor-adjacent adipose (4IC), metaplastic TNBC tumor control adipose (4IC), non-metaplastic TNBC tumor-adjacent adipose (4M4), and non-metaplastic TNBC control adipose samples. Due to processing issues and low sample availability, the 4M4 tumor was unable to be imaged. All samples were fixed overnight in a formaldehyde acetic acid (FAA) solution at room temperature. The samples were then dehydrated using a series of 30 min ethanol washes with increasing concentrations (30%, 50%, 70%, 90%, and 100%) over a span of two days. Critical point drying was used to remove water with zero surface tension and prevents collapse of fibers and pores. Following critical point drying, samples were then sputtered with platinum coating. SEM images were taken at 10 kX, 25 kX, and 50 kX at three separate sites using a Quanta™ 3D DualBeam™ FEG FIB-SEM (LSU Shared Instrument Facility, Baton Rouge, LA, USA) with settings at 5 kV and 3 pA. Assessment of ECM fiber characteristics was performed using previously published methods [25,26]. Briefly, SEM images of decellularized biospecimens were first baseline-adjusted to express the lowest grayscale pixel to 0, or true black, using the FIJI adaptation feature of the ImageJ software (2.0.0-rc-71) [27,28]. After classifying and segmenting the images with Weka Trainable Segmentation tool [29] to distinguish fibers from fiber edges, the FIJI plugins DiameterJ and OrientationJ (v1.018) were used to generate data on the distributions of pore size, fiber orientation, and fiber diameter. The number of black pixels was used to measure and quantitate pore size (*n* = 3 images). To determine fiber orientation, at each pixel, OrientationJ was used. OrientationJ was used with standard angular histogram settings, and fiber orientations were calculated by first generating a centerline over each identified fiber. Fiber orientation normalization was then performed to compare orientations across all fibers as their angles were in reference to the reference line produced by OrientationJ, following previously published methods [25]. A total of 180 bins were applied in the OrientationJ angular distribution analysis, giving a resolution of 1° per bin. Orientation indexes are available as Supplemental Table S1, where 0 values represent random fibers and values of 1 represent aligned fibers. To measure fiber diameter (*n* = 3 images), a Euclidean distance transformation algorithm was used to assign a grayscale value to each fibrous pixel equal to that pixel’s orthogonal distance to the nearest pore [30].

### 2.12. Oscillating Rheometry

Oscillating rheometry testing was performed on decellularized tumor-adjacent and control adipose tissues for both 4IC and 4M4 tissue samples. Sample pieces were measured and cut at a relative thickness of 1–2 mm and biopsy punched using an 8 mm biopsy punch to normalize sample pieces. If a sample deviated in size after decellularization, that sample was not included in testing to maintain normalization. Testing was performed using a Discovery HR-2 Hybrid Rheometer (TA Instruments, New Castle, DE, USA) with an 8 mm parallel plate. Storage (G′) and loss (G′′) moduli were determined by frequency sweeps from 0.628 to 62.8 (rad/s) and a strain of 10%. 4IC samples were tested in duplicate using two separate pieces. However, due to limited sample availability, 4M4 sample testing was unable to be performed in replicates. To compare stiffness properties between the two donors, the storage modulus was compared at 1 Hz, or where frequency independence was observed.

### 2.13. Pathway Analysis and MFAP2 Expression Data Mining

Unbiased pathway analysis was performed through Enrichr utilizing the MSigDB Hallmark 2020 pathway in Enrichr [20,21,31]; the access date was October 2025. Genes included in the analysis were all significantly changed genes with an adjusted *p*-value of *p* < 0.05. MFAP2 gene expression and correlation maps were generated from publicly available RNA sequencing data using the BC-GenExMiner (v5.2) [32]. For MFAP2 expression across breast cancer subtypes, *n* = 4421 patients were evaluated from three RNA-seq data sets: TCGA, SCAN-B GSE96058, and SCAN-B GSE81538. Targeted expression analysis of MFAP2 criteria included all intrinsic molecular subtypes, and splitting criteria were applied for PAM50 subtypes. The access date was December 2025. Unbiased correlation pathways utilized Ontologies, GO: Biological Processes data set. The access date was October 2025.

### 2.14. Statistical Analysis

Statistical analyses were performed using GraphPad Prism (v4). One-way ANOVA was performed to determine statistical significance of pore size and fiber attributes, unpaired Student’s *t*-test was used to assess oscillating rheometry, and two-way ANOVA was performed for drug response. *p*-values < 0.05 were considered statistically significant.

Generative artificial intelligence (GenAI) was not used in the design or writing of this study.

## 3. Results

### 3.1. Metaplastic TNBC Tumor and Tumor-Adjacent Adipose Tissue Has Altered ECM Architecture

**Figure 1:**
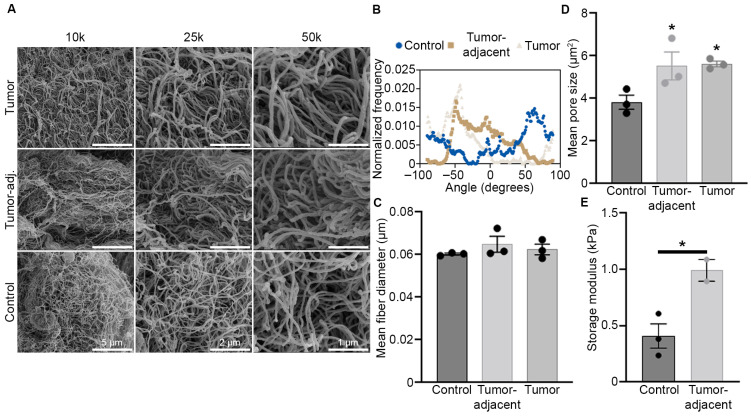
Metaplastic TNBC has increased pore size and matrix stiffness. (**A**) Representative SEM images of decellularized primary metaplastic TNBC, tumor-adjacent adipose tissue, and control adipose tissue (opposite breast) of the tumor. Three separate regions were imaged at 10 kX, 25 kX, and 50 kX. (**B**) Relative fiber orientation of decellularized primary metaplastic tumor, tumor-adjacent tissue, and control adipose tissue as obtained from SEM imaging. Fiber orientation is on the *x*-axis and distribution of fibers at each angel is shown on the *y*-axis, where 0 values represent random fibers and values of 1 represent aligned fibers. (**C**) Relative fiber diameter derived from SEM imaging and calculated in ImageJ, *n* = 3 separate imaged regions ± SEM. (**D**) Mean matrix pore size in decellularize metaplastic tumor, tumor-adjacent tissue, and control adipose tissue. Calculations derived from SEM images taken at 25 K magnification. *n* = 3 separate imaged regions ± SEM. (**E**) tumor-adjacent and control adipose samples were evaluated through a frequency sweep, using oscillating rheology. At 1 Hz, storage modulus was compared. Asterisks indicate statistical difference (*p* < 0.05) from control adipose tissue. *n* = 3 (control adipose) and *n* = 2 (tumor-adjacent) spatially different samples ± SEM. One-way ANOVA was performed to determine statistical significance of pore size and fiber attributes, while unpaired Student’s *t*-test was used to assess oscillating rheometry.

Studies on matrix remodeling in breast cancer have reported changes in collagen I expression, fiber alignment at the tumor/stromal interface, and overall tumor stiffness [5,7,33,34,35,36,37]. However, to date there has not yet been a comprehensive evaluation of ECM transformation (composition, fiber alignment, stiffness) in the metaplastic TNBC subtype. To gain greater insight on ECM remodeling in metaplastic TNBC, we evaluated a primary metaplastic triple negative tumor, TU-BcX-4IC (4IC), compared to breast tissue adjacent to the tumor (tumor-adjacent) and normal breast tissue taken from the opposite side of the same breast (control adipose). To increase visualization of ECM, tissue samples were decellularized, and matrix architecture was assessed with SEM. Alterations in metaplastic ECM were evaluated for ECM fiber alignment, fiber thickness, and porosity. SEM imaging revealed random fiber alignment in all three tissue types (Figure 1A,B). This was further confirmed through analysis of fiber orientation and calculated orientation index (Supplemental Table S1). Tumor, tumor-adjacent, and control adipose ECM fibers were visually similar in diameter, corroborated by evaluation of fiber thickness using DiameterJ software, showing no statistical differences among metaplastic tumor, tumor-adjacent adipose, and control adipose (Figure 1C). The average pore size of the metaplastic tumor was significantly larger (5.62 µm^2^ ± 0.19 µm^2^, *p* < 0.05) than that of the control adipose tissue (3.38 µm^2^ ± 0.47 µm^2^), but comparable in size to the tumor-adjacent tissue (5.53 µm^2^ ± 0.93 µm^2^, *p* > 0.05) (Figure 1D). In accordance with this, tumor-adjacent tissue pore size was significantly larger than that of control adipose tissue (*p* < 0.05). Differences in matrix stiffness between only tumor-adjacent and control adipose tissues were next evaluated by oscillating rheometry, as sufficient tumor tissue was not available for analysis. Tumor-adjacent tissue had higher storage modulus (G’) and storage loss (G”) values compared to control adipose tissue (Figure 1E). Based on available samples, tumor-adjacent tissue appeared stiffer than control adipose tissue, measuring at 990.41 Pa and 407.82 Pa.

### 3.2. Metaplastic TNBC Is Enriched with Glycoproteins, Minor Fibrillar Collagens, and Fibril-Associated Collagens

**Figure 2:**
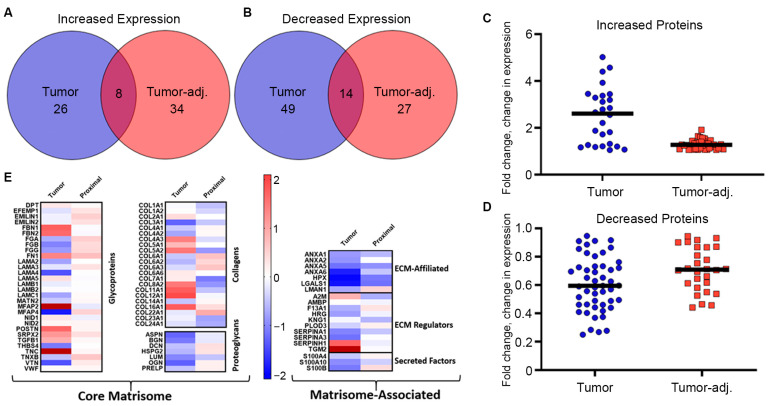
Metaplastic TNBC is enriched for glycoproteins and non-fibril collagens. (**A**–**D**) Primary metaplastic TNBC tumor, tumor-adjacent breast tissue, and control adipose breast tissue from the same patient were decellularized and evaluated via proteomics for differences in matrix protein expression. Proteins were identified that were commonly (**A**) upregulated and (**B**) downregulated in tumor and tumor-adjacent adipose normalized to parental controls. (**C**,**D**) Graphical representation of total protein changes in tumor and tumor-adjacent breast tissue. (**E**) Heat map representation of proteins altered between primary tumor, tumor-adjacent breast tissue, and control adipose tissue. Red bars represent logfold increased expression and blue bands represent decreased logfold expression.

To determine protein changes in the metaplastic TNBC ECM, decellularized tumor, tumor-adjacent adipose, and control adipose samples were evaluated through proteomics. Expression in control adipose tissue was used as a baseline to measure fold change differences in both the tumor and tumor-adjacent samples. Compared to control adipose breast tissue, there were 34 total upregulated proteins and 63 total downregulated proteins in the TU-BcX-4IC metaplastic tumor (Figure 2A–D, Table 1). The tumor-adjacent adipose tissue had 42 total proteins upregulated and 41 proteins downregulated compared to the control adipose tissue. Primary metaplastic tumor and tumor-adjacent adipose tissue similarly regulated a total of 22 proteins compared to control adipose tissue, with 8 of these proteins (TNC, COL16A1, POSTN, FN1, SRPX2, TGFBI, NID2, VWF) demonstrating elevated expression and 15 of these proteins (KNG1, COL23A1, COL24A1, HRG, ANZA1, S100B, LUM, SERPINA1, BGN, ANXA5, ASPN, COL3A1, ANXA6, LGALS1, HPX) demonstrating decreased expression. While both the metaplastic tumor and tumor-adjacent adipose tissue had comparable relative numbers of observed proteins changed, expression of proteins in the metaplastic tumor exhibited a higher fold-change compared to less robust changes observed in the tumor-adjacent adipose (Figure 2C,D). In addition, there were ten proteins upregulated in the metaplastic tumor that were downregulated in the tumor-adjacent adipose tissue (MFAP2, COL12A1, COL11A1, SERPINH1, COL5A2, COL8A2, A2M, NID1, COL7A1) (Table 1). Inversely, there are 24 proteins downregulated in the metaplastic TNBC tumor but upregulated in the tumor-adjacent tissue (EMILIN1, EMILIN2, MFAP4, FGA, FGB, FGG, VTN, COL14A1, COL22A1, COL6A1, COL6A2, COL6A3, COL4A1, DCN, HSPG2, OGN, PRELP, LAMN1, F13A1, PLOD3, TNXB, LAMA3, EFEMP1) (Table 1).

Evaluation of matrix classifications for altered proteins suggest that protein changes occurred primarily in adhesive and matrix organizational glycoproteins, fibril-associated and minor fibrillar collagens, and ECM regulators (Figure 2E). Microfibrillar-associated protein 2 (MFAP2) and several other glycoproteins (TNC, FBN1, FBN2, POSTN, FN1, SRPX2) showed the largest increases in expression (Table 1). Collagen proteins that demonstrated the highest increase in expression in the metaplastic tumor compared to control adipose tissue included fibril-associated collagens (FACIT) (COL12A1, COL16A1, COL5A1, COL5A2, COL11A1), hexagonal network-forming collagens (COL8A2), and basement membrane collagen (COL4A3). In this sample of the metaplastic TNBC tumor, there was no change in major fibril collagen, COL1A1 and COL1A2, and decreased expression for COL3A1. Tumor-adjacent tissue exhibited decreased expression for COL1A1, COL1A2, and COL3A1. Collectively, expression of filamentous collagen chains commonly associated with adipose tissues were decreased (COL6A1, COL6A2, COL6A3) (Table 1). Protein expression of glycoproteins, fibril-associated collagens, and filamentous collagens was enriched in the ECM of adipose tissue adjacent to the metaplastic tumor (Table 1). ECM organizational proteins (TNXB, FGA, FGB) and elastic-associated proteins (EMILIN1, MFAP4) were also elevated in tumor-adjacent adipose tissue. Collagens with the highest levels of expression in tumor-adjacent tissue included fibril-associated collagens (COL16A1, COL22A1) and filamentous collagens (COL6A1, COL6A2, COL6A3). Proteomics of tumor-adjacent adipose tissue compared to control adipose tissue demonstrated a decrease in expression for major fibrillar collagens (COL1A1, COL1A2, COL3A1), minor fibrillar collagens (COL5A2, COL11A1, COL24A1), basement membrane collagen (COL4A3), hexagonal network-forming collagen (COL8A2), and anchoring fibril collagen COL7A1 (Table 1).

### 3.3. Glycoprotein, MFAP2, Elevated in Metaplastic TNBC Correlates with Poor Patient Survival

**Figure 3:**
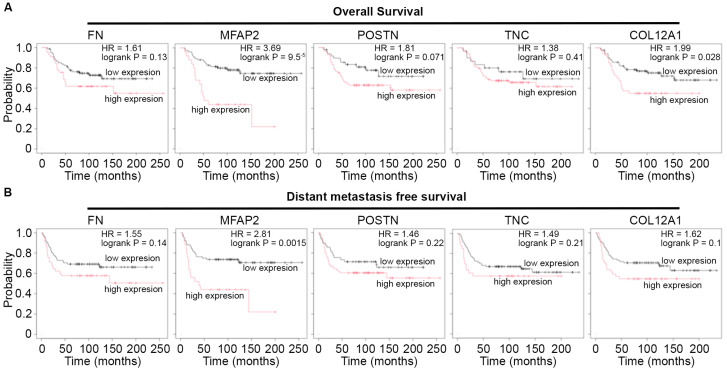
MFAP2 correlates with poor patient outcomes in TNBC. (**A**,**B**) Kaplan–Meier plot for patient survival for proteins elevated in metaplastic TNBC compared to control adipose tumor. KM plots were generated for (**A**) overall patient survival and (**B**) distant metastasis-free survival. Results derived from *n* = 126 distinct tumors negative for estrogen receptor, progesterone receptor, and HER2. Access date October 2025.

To determine the potential impact of the observed ECM changes in glycoproteins and less described collagens on patient survival, Kaplan–Meier (KM) analysis was performed [24] for ECMs elevated in the metaplastic tumor. Among proteins with available data in receptor-negative breast tumors, KM plots were generated for correlations of protein expression for glycoproteins (FN1, MFAP2, POSTN, TNC) and collagens (COL12A1) relative to overall patient survival and distant metastasis-free survival (Figure 3). Notably, only high expression of the glycoprotein MFAP2, which exhibited the greatest upregulated fold change in the metaplastic TNBC tumor compared to control adipose tissue, had protein expression associated with decreased patient overall survival and distant metastasis-free survival (Figure 3).

**Figure 4:**
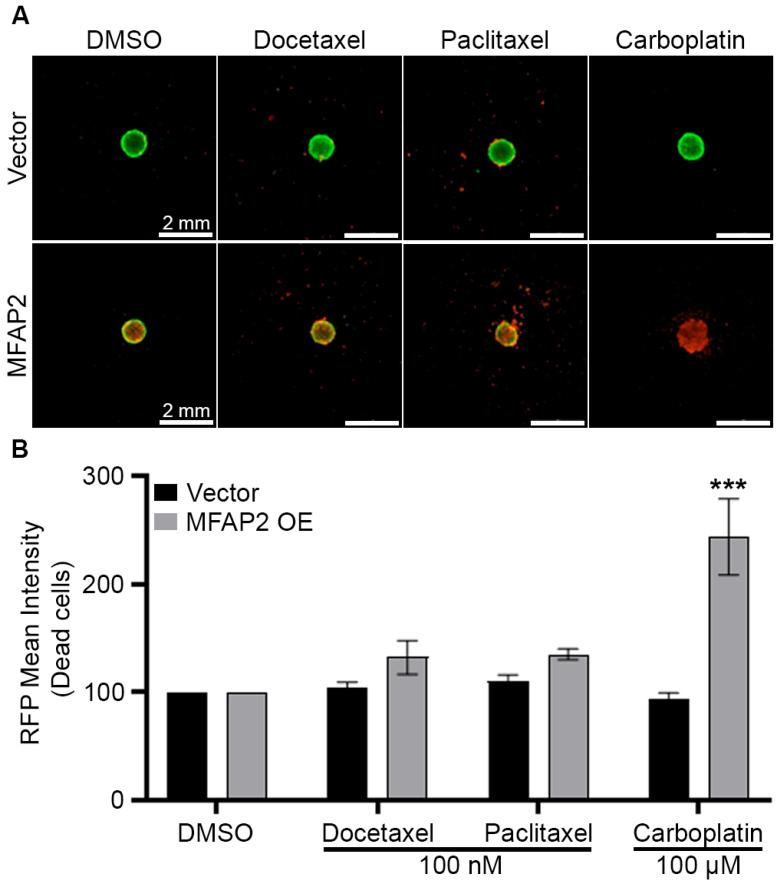
MFAP2 over expression sensitizes metaplastic TNBC to chemotherapy. TU-BcX-4IC-MFAP2 OE and vector cell lines were seeded at 5000 cells/spheroid and treated with escalating doses of chemotherapy (docetaxel, paclitaxel, carboplatin) for 72 h. (**A**) Representative images of LIVE/DEAD stain for fluorescent calcein-AM (live) and ethidium homodimer-1 (dead) cells at endpoint. (**B**) Graphical representation of ethidium intensity representing dead cells. Normalization was performed on vehicle control and error bars represent SEM. *n* = 3 biological replicates. *** Significant difference *p* < 0.001. To determine statistical significance, two-way ANOVA Sidak’s multiple comparisons test was performed.

To further assess the role of MFAP2 in breast cancer biology and regulation of the metaplastic TNBC phenotype, we overexpressed MFAP2 (MFAP2 OE) in a primary metaplastic TNBC cell line (TU-BcX-4IC) and non-metaplastic TNBC cell line (MDA-MB-231) (Supplemental Figure S2A,B). TU-BcX-4IC-MFAP2 cell lines were next screened at escalating doses (0.1–100 nM and 0.1–100 mM) of standard of care neo-adjuvant chemotherapies (NACT) (docetaxel, paclitaxel, carboplatin) in 2D and 3D. There was no difference in sensitivity to chemotherapy response in either TU-BcX-4IC-MFAP2 cell lines compared to vehicle control in 2D (Figure 3A,B). The 3D screens for changes in MFAP2-mediated therapeutic response demonstrated a significant increase (243.5% ± 35.08 SEM, *p* < 0.001) in sensitivity to high doses (100 nM) of Carboplatin in the TU-BcX-4IC-MFAP2 cell line (Figure 4).

**Figure 5:**
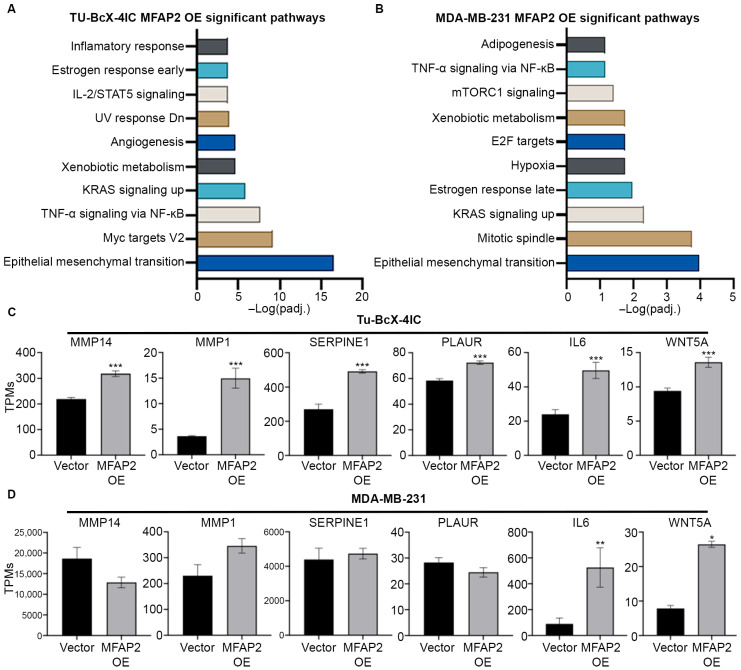
MFAP2 enhances an epithelial-to-mesenchymal gene signature in metaplastic TNBC cells. RNA sequencing was performed on TU-BcX-4IC and MDA-MB-231-vector and -MFAP2 overexpressing cell lines. Total gene changes were identified in OE cell lines normalized to vector controls. Pathway analysis of RNA sequencing for pathways associated with significant upregulated genes in (**A**) TU-BcX-4IC-MFAP2 and (**B**) MDA-MB-231-MFAP2 cells. Pathway analysis was obtained from Enrichr database utilizing MSigDB Hallmark 2020 pathway. Gene changes in EMT-associated genes in (**C**) TU-BcX-4IC-MFAP2 and (**D**) MDA-MB-231-MFAP2 cell lines. Results represent gene expression as TPMs. *n* = 3 biological replicates. Error bars represent SEM. * Significant difference padj < 0.05, ** significant difference padj < 0.01, *** significant difference padj < 0.001. Differentially expressed genes determined by DESeq2.

As MFAP2 expression did not significantly increase resistance of metaplastic TNBC response to NACT treatment, we analyzed transcriptomic changes induced by MFAP2 expression in metaplastic TNBC and non-metaplastic TNBC cells. Through RNA sequencing analysis, we observed enhanced expression of genes associated with pathways linked to EMT (Tu-BcX-4IC MFAP2 OE: p_adj_ = 3.34 × 10^−17^; MDA-MB-231 MFAP2 OE: p_adj_ = 0.000103) and inflammatory signaling pathways such as TNF-a signaling (Tu-BcX-4IC MFAP2 OE: p_adj_ = 2.35 × 10^−8^) and IL-2/STAT5 signaling (Tu-BcX-4IC MFAP2 OE: p_adj_ = 0.00017) in MFAP2-expressing cells compared to vector control. Both cell lines had elevated genes in these pathways; however, the 4IC-MFAP2 OE line demonstrated a greater degree of total genes changed associated with each pathway (Figure 5). Compared to vector control, TU-BcX-4IC-MFAP2 OE cells had significantly higher levels of matrix metalloproteases 14 and 1 (MMP14 and MMP1), SERPINE1, PLAUR, IL6, and WNT5A (Figure 5C). In contrast, only expression of IL6 and WNT5A was enhanced in the MDA-MB-231-MFAP2 OE cell line compared to the vector control (Figure 5D).

### 3.4. MFAP2 Is Associated with Matrix Remodeling and Cancer Stem Cell (CSC) Phenotype in TNBC

**Figure 6:**
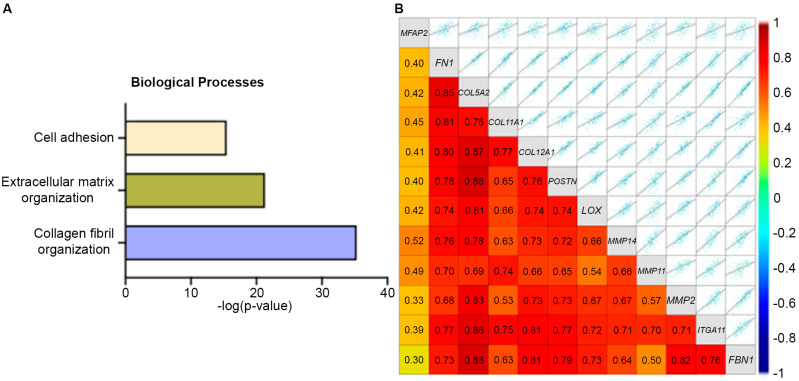
MFAP2 expression is associated with EMT profile and ECM organization. (**A**) Unbiased pathway map for GO biological processes for genes positively correlated with MFAP2 expression in TNBC. (**B**) Correlation map for gene expression of MFAP2-associated genes specific to EMT/invasion and matrix remodeling. Pathway and correlation maps were generated form publicly available RNA sequencing data with the BC-GenExMiner (v5.2) for TNBC. Access date October 2025.

To further define the impact of MFAP2 in breast cancer, MFAP2 expression in the different breast cancer subtypes was evaluated and unbiased correlation mapping of MFAP2 expression in TNBC/Basal like tumors was performed. Results demonstrated that MFAP2 expression was significantly higher in Basal-like tumors compared to HER2 amplified (*p* < 0.01), luminal B (*p* < 0.0001), and normal-like tumors (*p* < 0.001). Expression of MFAP2 in Basal-like tumors was equal to that of luminal A tumors (*p* < 0.1) (Supplemental Figure S4A). Evaluation of correlation mapping demonstrated that genes positively correlated with MFAP2 expression were highly linked to the Collagen Fibril Organization, Extracellular Matrix Organization, and Cell Adhesion Biological processes in TNBC (Figure 6A). Interestingly, many genes that positively correlated with MFAP2 expression were also elevated in the metaplastic tumor proteomics (Figure 6B), in addition to genes associated with matrix stiffening (LOX), cell invasion (MMPs), and a cancer stem cell phenotype (ITGA11) [38]. Similar trends in MFAP2 expression were also observed in the other breast cancer subtypes (Supplemental Figure S4B).

**Figure 7:**
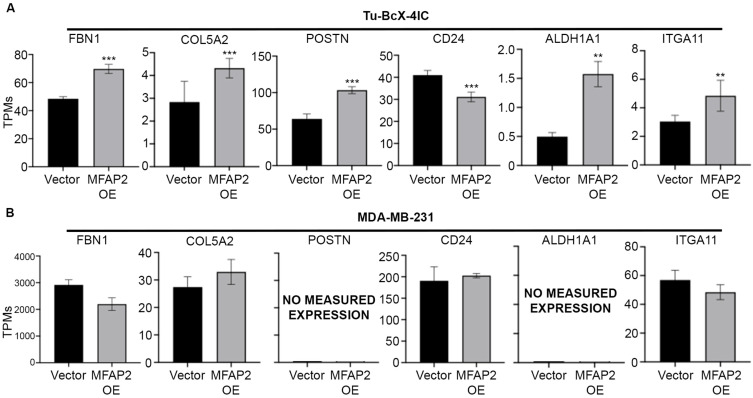
MFAP2 expression induces a metaplastic ECM signature and CSC gene signature in metaplastic TNBC cells. Gene expression for (**A**) ECM genes and (**B**) CSC-associated genes as obtained from RNA sequencing in TU-BcX-4IC and MDA-MB-231-vector and -MFAP2 overexpressing cell lines. Results represent gene expression as TPMs. *n* = 3 biological replicates. Error bars represent SEM. ** significant difference *p* < 0.01, *** significant difference *p* < 0.001. Differentially expressed genes determined by DESeq2.

Evaluation of exogenous MFAP2 transcriptome changes revealed upregulated expression of previously published metaplastic-associated ECM genes (FBN1, COL5A2, POSTN) and cancer stem cell signature ALDH1A1 and ITGA11, and repressed CD24, suggesting MFAP2 expression was associated with increased CSC-associated markers (Figure 7A) [16]. Similar fold changes in gene expression were not detected in the non-metaplastic TNBC MFAP2 OE cell line compared to the vector control for these genes (Figure 7B).

## 4. Discussion

Metaplastic breast cancer is a rare and aggressive subtype of breast cancer characterized by unique histological features, poor response to conventional therapies, and poor survival outcomes [16]. While metaplastic tumors account for only 1% of all breast tumors, they are disproportionately aggressive, having a worse prognosis than TNBC, with higher rates of recurrence, metastasis, and limited response to therapeutics [16]. Recent evidence suggests that metaplastic breast tumors show extensive ECM remodeling, including altered protein expressions and increased stiffness [39]. This change in the TME warrants increased investigation to better guide targeted treatments and improve patient outcomes [40].

We sought to characterize the ECM architecture, stiffness, and protein expression of a metaplastic tumor. Once characterized, these ECM modification could be exploited for accurate tumor model development and therapeutic targeting. The increased pore size observed in metaplastic TNBC tumors and adjacent adipose tissue (Figure 1) reveals ECM changes that have been reported to enhance cell motility, invasiveness, and paracrine signaling [41,42]. To our knowledge, this is the first study to evaluate pore size in a metaplastic TNBC tumor compared to matched control adipose tissue. These structural shifts in ECM pore structure are suggested to promote EMT and metastasis, offering potential insight into the aggressive behavior of metaplastic TNBC [43]. Rheometry results revealed increased stiffness in tumor-adjacent tissue compared to the control. Although direct tumor testing was not feasible in this study, elevated ECM proteins including FN1, POSTN, TNC, and collagens (Figure 2) suggest an increase in stiffness and warrant further investigation. Mechanical shifts in ECM can promote tumorigenesis and metastasis through altered mechano-transduction and ECM architecture [44,45,46,47]. These preliminary findings support the need to mimic ECM architecture for more physiologically relevant in vitro modeling.

Breast cancer alters ECM protein composition, often increasing fibrillar collagens I and III [48]. While collagen I is widely used to model tumor progression, it is not observed to be elevated across all breast cancer subtypes, and low collagen I expression in breast cancer is associated with TNBC, node-positive, and high-grade tumors [7]. To better model metaplastic TNBC, we characterized the ECM protein expression of a metaplastic tumor. Our findings demonstrated that this metaplastic TNBC lacked enrichment of collagen I or III (Figure 2). This suggests metaplastic breast tumors may possess an ECM profile that is distinct from other subtypes. Further, when compared to prior work on non-metaplastic TNBC [6], the decellularized samples identified proteins not detected in non-metaplastic TNBC tumors, suggesting potential subtype-specific ECM features. While some differences in proteomic assessment may stem from using native tissue vs. decellularized tissue, our findings identified 25 proteins with similar expression, and 38 unique proteins, potentially highlighting how decellularization can uncover low-abundance ECM proteins.

Proteomic data demonstrated an ECM protein composition in tumor-adjacent tissue that was distinct from what was observed in the tumor tissue. Spatial heterogeneity may contribute to the observed discrepancies; however, it should be noted that tumor-adjacent tissue demonstrated a specific enrichment of hemostatic associated glycoproteins (FGA, FGB, FGG, VTN) (Table 1), all of which are involved in providing a provisional matrix during normal wound healing [49]. Tumors are described as wounds that never heal, the enrichment of these clotting factors in the tumor-adjacent tissue may represent the stromal response to wound signals from the tumor [50]. Proteins that were enhanced in both the metaplastic tumor and tumor-adjacent adipose tissue (FN1, POSTN, TGFBI, TNC, COL16A1) (Figure 1A) were association with both EMT and metastatic potential. While additional studies are required with enhanced sample numbers, this pilot study suggests a potential mechanism by which both the tumor and adjacent adipose tissue remodel to support a more aggressive phenotype.

Specifically, within metaplastic breast cancer, the overexpression of glycoproteins and collagens including MFAP2, FN1, POSTN, TNC, COL5A2, COL6A6, and COL11A1 have all been reported to be upregulated in aggressive, metastatic breast cancer subtypes [51,52,53,54,55,56]. COL11A1 and FBN2 have previously been described as elevated in metaplastic TNBC compared to non-metaplastic TNBC [15]. Further, we demonstrate the ECM protein MFAP2 as a mediator of MMP gene expression (Figure 5) and MMPs are elevated in metaplastic TNBC compared to non-metaplastic TNBC [15]. Collectively, we suggest that MFAP2 expression induces enrichment for MMP gene expression and creates a microenvironment which favors metastatic potential. Moreover, Kaplan–Meir plots of overexpressed ECM proteins show significant association with poor patient overall survival and distant metastasis-free survival with high MFAP2 protein expression (Figure 3). In accordance with this, prior works on MFAP2 have highlighted a role for MFAP2 in cancer progression, specifically through increased EMT, cell motility, and invasion [57,58]. While established in other cancer types, few studies have focused on the impact of MFAP2 and breast cancer. One prior work has suggested a role for MFAP2 in breast cancer progression through increased invasion [59] and demonstrated elevated MFAP2 gene expression in breast tumors compared to normal tissue [59]. The suggested mechanistic action of MFAP2 is through TGFβ and WNT signaling cascades [57]. Our transcriptomic analysis demonstrated elevated WNT signaling factor WNT5A (Figure 5) in both the TU-BcX-4IC-MFAP2 and MDA-MB-231-MFAP2 OE cell lines, further suggesting MFAP2 as a regulator of WNT signaling in breast cancer. Recent studies have linked non-canonical WNT signaling factors (WNT5B, WNT5A) to mesenchymal breast cancer cells [60] and breast cancer subtypes with high incidences of metastasis [61]. While a link to MFAP2 and cancer outcomes is observed, to date, MFAP2 is not a prognostic marker for outcomes in breast cancer. Further investigation into the prognostic value of MFAP2 expression and additional ECM modifications, such as porosity and fiber alignment, is warranted, as recent works have revealed ECM structure and composition as a potential prognostic marker. Tumor-associated collagen signatures are suggested as a prognostic indicator in breast cancer [62,63], ECMs (collagen, fibronectin, elastin) are linked to breast cancer progression, outcomes, and response to therapy [7,64,65,66,67], and matrix composition mediates therapeutic response in vitro [5]. Further, our results demonstrate tumor tissue as retaining a random fiber orientation and increased porosity (Figure 1); to date, the bulk of in vitro research on breast cancer and fiber orientation has focused on aligned fibers and the induction of EMT and cell motility [68,69]. While porosity has been identified in vitro as a mediator of cancer cell motility, proliferation, and cancer stem phenotype [70,71], the prognostic impact of porosity is not defined. Preclinical models that can accurately mimic the random fiber orientations, porosity, and ECM composition of distinct breast cancer subtypes would enhance our understanding of cell extrinsic mediators or breast cancer progression and intracellular signaling.

Limits to this study include the sample size of the metaplastic tumor and matched tissue (*n* = 1). Metaplastic breast cancer is a rare cancer, occurring in less than 1–5% of breast cancer patients [16,17]; this study was designed as a pilot study to provide initial insight on the composition and structure of a metaplastic tumor. The ultimate intent of this work is to foster future studies aimed at validating metaplastic TNBC specific matrix compositions and aid in 3D tumor model development of this rare disease. Our method of decellularization in combination with proteomics required an ample supply of matrix protein that created additional limiting factors. There is a lack of experimental studies dedicated to elucidating less described ECM proteins, specifically upregulated minor fibrillar and fibril-associated collagens, that could have an impact on cancer progression and drug resistance. Hence, our proteomics evaluation highlights the need to identify these less described proteins that are enriched in metaplastic and triple-negative tumors. We acknowledge the limitations imposed by using dissected pieces cut from decellularized tissues; while decellularization may increase depth in discovery of less identified ECMs, decellularization itself may result in loss of protein, future comprehensive studies should integrate both native and decellularized biospecimens. Breast tissue may display inherent variability between distinct regions of the breast; when working with limited overall sample amounts and a single donor sample, additional limitation may arise. Such limitations include limited imaging fields, tissue heterogeneity driving differences versus true spatial differences, and the inability to control analytical testing of a precise area. Additionally, while oscillating rheology provided a bulk analysis of tissue stiffness, additional methods such as AFM would provide information on local matrix stiffness and provide information on mechanosensing at the cellular level. Further experimentation that allows for the measuring of multiple controlled regions and additional donors would give a firm representative idea of ECM structural characteristics.

## 5. Conclusions

Our major findings offer novel insights into the biomechanical and biochemical properties of a metaplastic TNBC. The results here suggest a matrix-specific subtype that must be critically considered when using in vitro modeling for experimentation. Furthermore, the recent paradigm shifts by the NIH, FDA, and other federal agencies away from animal models further reinforces the critical need for advanced 3D tumor microenvironment modeling approaches to create in vitro platforms that better recapitulate in vivo disease progression. Although our results provide insight into what to expected when evaluating metaplastic TNBC ECM, continued investigations using additional metaplastic and non-metaplastic TNBC are warranted to further guide 3D modeling.

## Figures and Tables

**Table 1:** ECM protein changes in metaplastic TNBC tumor and tumor-adjacent breast tissue.

Protein Type	Tumor		Tumor-Adjacent	
	Upregulated	Downregulated	Upregulated	Downregulated
**Glycoproteins**	DPT, FBN1, FBN2, FN1, LAMA5, MFAP2, NID1, NID2, POSTN, SRPX2, TGFBI, TNC, VWF	EFEMP1, EMILIN1, EMILIN2, FGA, FGB, FGG, LAMA2, LAMA3, LAMA4, LAMB1, LAMC1, MATN2, MFAP4, THBS4, TNXB, VTN	EFEMP1, EMILIN1, EMILIN2, FGA, FGB, FGG, FN1, LAMA3, MATN2, MFAP4, NID2, POSTN, SRPX2, TGFBI, TNC, TNXB, VTN, VWF	MFAP2, NID1
**Collagens**	COL2A1, COL4A3, COL5A1, COL5A2, COL6A6, COL7A1, COL8A2, COL11A1, COL12A1, COL16A1	COL3A1, COL4A1, COL4A2, COL6A1, COL6A2, COL6A3, COL14A1, COL22A1, COL23A1, COL24A1	COL4A1, COL6A1, COL6A2, COL6A3, COL14A1, COL16A1, COL22A1	COL1A1, COL1A2, COL3A1, COL4A3, COL5A2, COL7A1, COL8A2, COL11A1, COL12A1, COL23A1, COL24A1
**Proteoglycans**		ASPN, BGN, DCN, HSPG2, LUM, OGN, PRELP	DCN, HSPG2, OGN, PRELP	ASPN, BGN, LUM
**Matrix-** **Associated**	A2M, SERPINH1, TGM2	ANXA1, ANXA2, ANXA5, F13A1, HPX, HRG, KNG1, LGALS1, LMAN1, PLOD3, S100A10, S100A4, S100B, SERPINA1, SERPINA3	LMAN1, F13A1, PLOD3, S100A10, S100A4	A2M, ANXA1, ANXA5, ANXA6, HPX, HRG, KNG1, LGALS1, S100B, SERPINA1, SERPINH1

## Data Availability

All data are contained within this manuscript and the supplemental documents. The 4IC RNA sequencing data discussed in this publication have been deposited in NCBI’s Gene Expression Omnibus, accessible through GEO Series accession number (GSE314824).

## Supplementary Materials

The following supporting information can be downloaded at: https://www.mdpi.com/article/10.3390/bioengineering13010047/s1, Supplemental Figure S1: Fiber analysis of TNBC control adipose and tumor-adjacent breast tissue, Supplemental Figure S2: Confirmation of MFAP2 overexpression, Supplemental Figure S3: 2D and 3D therapy response in MFAP2 overexpressing breast cancer cell lines, Supplemental Figure S4: MFAP2 expression across correlated expression with EMT and matrix genes across breast cancer subtypes, Supplemental File S1: Proteomics data of metaplastic tumor, tumor-adjacent adipose and control adipose, Supplemental File S2: RNA sequencing of MFAP2 OE cell lines, Supplemental Table S1: OI for SEM imaging and fiber alignment.

## Abbreviations

The following abbreviations are used in this manuscript:

ECM	Extracellular matrix
EMT	Epithelial-to-mesenchymal transition
MMP	Matrix metalloproteinase
TNBC	Triple negative breast cancer
4IC	TU-BcX-4IC
MFAP2	Microfibril associated protein 2
SEM	Scanning electron microscopy
CSC	Cancer stem cell

## References

[1] Infanger D.W., Lynch M.E., Fischbach C. (2013). Engineered culture models for studies of tumor-microenvironment interactions. Annu. Rev. Biomed. Eng..

[2] Lee J.J., Ng K.Y., Bakhtiar A. (2025). Extracellular matrix: Unlocking new avenues in cancer treatment. Biomark. Res..

[3] Lu P., Takai K., Weaver V.M., Werb Z. (2011). Extracellular matrix degradation and remodeling in development and disease. Cold Spring Harb. Perspect. Biol..

[4] Belgodere J.A., King C.T., Bursavich J.B., Burow M.E., Martin E.C., Jung J.P. (2018). Engineering Breast Cancer Microenvironments and 3D Bioprinting. Front. Bioeng. Biotechnol..

[5] Byrne C.E., Decombe J.B., Bingham G.C., Remont J., Miller L.G., Khalif L., King C.T., Hamel K., Bunnell B.A., Burow M.E. (2021). Evaluation of Extracellular Matrix Composition to Improve Breast Cancer Modeling. Tissue Eng. Part A.

[6] Tang W., Zhou M., Dorsey T.H., Prieto D.A., Wang X.W., Ruppin E., Veenstra T.D., Ambs S. (2018). Integrated proteotranscriptomics of breast cancer reveals globally increased protein-mRNA concordance associated with subtypes and survival. Genome Med..

[7] Jansson M., Lindberg J., Rask G., Svensson J., Billing O., Nazemroaya A., Berglund A., Wärnberg F., Sund M. (2024). Stromal Type I Collagen in Breast Cancer: Correlation to Prognostic Biomarkers and Prediction of Chemotherapy Response. Clin. Breast Cancer.

[8] Dinkel Z., Baker A., Akins A., King K.L., Harrington M., Kunkel D., Gao Z., Ye T., Dunn H. (2025). Collagen architecture in triple negative breast cancer. PLoS ONE.

[9] Li F., Wei Y., Li L., Chen F., Bao C., Bu H., Zhang Z. (2025). Collagen Density Is Associated With Pathological Complete Response to Neoadjuvant Chemotherapy in Triple-Negative Breast Cancer Patients. J. Surg. Oncol..

[10] Mouw J.K., Ou G., Weaver V.M. (2014). Extracellular matrix assembly: A multiscale deconstruction. Nat. Rev. Mol. Cell Biol..

[11] Werfel J., Krause S., Bischof A.G., Mannix R.J., Tobin H., Bar-Yam Y., Bellin R.M., Ingber D.E. (2013). How changes in extracellular matrix mechanics and gene expression variability might combine to drive cancer progression. PLoS ONE.

[12] Yu T., Di G. (2017). Role of tumor microenvironment in triple-negative breast cancer and its prognostic significance. Chin. J. Cancer Res..

[13] Jena M.K., Janjanam J. (2018). Role of extracellular matrix in breast cancer development: A brief update. F1000Research.

[14] Walker C., Mojares E., Del Rio Hernandez A. (2018). Role of Extracellular Matrix in Development and Cancer Progression. Int. J. Mol. Sci..

[15] Djomehri S.I., Gonzalez M.E., da Veiga Leprevost F., Tekula S.R., Chang H.Y., White M.J., Cimino-Mathews A., Burman B., Basrur V., Argani P. (2020). Quantitative proteomic landscape of metaplastic breast carcinoma pathological subtypes and their relationship to triple-negative tumors. Nat. Commun..

[16] Reddy T.P., Rosato R.R., Li X., Moulder S., Piwnica-Worms H., Chang J.C. (2020). A comprehensive overview of metaplastic breast cancer: Clinical features and molecular aberrations. Breast Cancer Res..

[17] Thapa B., Arobelidze S., Clark B.A., Xuefei J., Daw H., Cheng Y.C., Patel M., Spiro T.P., Haddad A. (2022). Metaplastic Breast Cancer: Characteristics and Survival Outcomes. Cureus.

[18] Matossian M.D., Chang T., Wright M.K., Burks H.E., Elliott S., Sabol R.A., Wathieu H., Windsor G.O., Alzoubi M.S., King C.T. (2022). In-depth characterization of a new patient-derived xenograft model for metaplastic breast carcinoma to identify viable biologic targets and patterns of matrix evolution within rare tumor types. Clin. Transl. Oncol..

[19] Matossian M.D., Elliott S., Van Hoang T., Burks H.E., Wright M.K., Alzoubi M.S., Yan T., Chang T., Wathieu H., Windsor G.O. (2021). NEK5 activity regulates the mesenchymal and migratory phenotype in breast cancer cells. Breast Cancer Res. Treat..

[20] Chen E.Y., Tan C.M., Kou Y., Duan Q., Wang Z., Meirelles G.V., Clark N.R., Ma’ayan A. (2013). Enrichr: Interactive and collaborative HTML5 gene list enrichment analysis tool. BMC Bioinform..

[21] Kuleshov M.V., Jones M.R., Rouillard A.D., Fernandez N.F., Duan Q., Wang Z., Koplev S., Jenkins S.L., Jagodnik K.M., Lachmann A. (2016). Enrichr: A comprehensive gene set enrichment analysis web server 2016 update. Nucleic Acids Res.

[22] Pashos N.C., Scarritt M.E., Eagle Z.R., Gimble J.M., Chaffin A.E., Bunnell B.A. (2017). Characterization of an Acellular Scaffold for a Tissue Engineering Approach to the Nipple-Areolar Complex Reconstruction. Cells Tissues Organs.

[23] King C.T., Matossian M.D., Savoie J.J., Nguyen K., Wright M.K., Byrne C.E., Elliott S., Burks H.E., Bratton M.R., Pashos N.C. (2022). Liver Kinase B1 Regulates Remodeling of the Tumor Microenvironment in Triple-Negative Breast Cancer. Front. Mol. Biosci..

[24] Gyorffy B., Lanczky A., Eklund A.C., Denkert C., Budczies J., Li Q., Szallasi Z. (2010). An online survival analysis tool to rapidly assess the effect of 22,277 genes on breast cancer prognosis using microarray data of 1,809 patients. Breast Cancer Res. Treat..

[25] Gurrala R., Byrne C.E., Brown L.M., Tiongco R.F.P., Matossian M.D., Savoie J.J., Collins-Burow B.M., Burow M.E., Martin E.C., Lau F.H. (2021). Quantifying Breast Cancer-Driven Fiber Alignment and Collagen Deposition in Primary Human Breast Tissue. Front. Bioeng. Biotechnol..

[26] Hoang V.T., Matossian M.D., Ucar D.A., Elliott S., La J., Wright M.K., Burks H.E., Perles A., Hossain F., King C.T. (2020). ERK5 Is Required for Tumor Growth and Maintenance Through Regulation of the Extracellular Matrix in Triple Negative Breast Cancer. Front. Oncol..

[27] Schindelin J., Arganda-Carreras I., Frise E., Kaynig V., Longair M., Pietzsch T., Preibisch S., Rueden C., Saalfeld S., Schmid B. (2012). Fiji: An open-source platform for biological-image analysis. Nat. Methods.

[28] Schneider C.A., Rasband W.S., Eliceiri K.W. (2012). NIH Image to ImageJ: 25 years of image analysis. Nat. Methods.

[29] Arganda-Carreras I., Kaynig V., Rueden C., Eliceiri K.W., Schindelin J., Cardona A., Sebastian Seung H. (2017). Trainable Weka Segmentation: A machine learning tool for microscopy pixel classification. Bioinformatics.

[30] Hotaling N.A., Bharti K., Kriel H., Simon C.G. (2015). DiameterJ: A validated open source nanofiber diameter measurement tool. Biomaterials.

[31] Xie Z., Bailey A., Kuleshov M.V., Clarke D.J.B., Evangelista J.E., Jenkins S.L., Lachmann A., Wojciechowicz M.L., Kropiwnicki E., Jagodnik K.M. (2021). Gene Set Knowledge Discovery with Enrichr. Curr. Protoc..

[32] Jézéquel P., Gouraud W., Ben Azzouz F., Guérin-Charbonnel C., Juin P.P., Lasla H., Campone M. (2021). bc-GenExMiner 4.5: New mining module computes breast cancer differential gene expression analyses. Database.

[33] Heydari S., Tajik F., Safaei S., Kamani F., Karami B., Dorafshan S., Madjd Z., Ghods R. (2025). The association between tumor-stromal collagen features and the clinical outcomes of patients with breast cancer: A systematic review. Breast Cancer Res..

[34] Xu R., Yin P., Wei J., Ding Q. (2023). The role of matrix stiffness in breast cancer progression: A review. Front. Oncol..

[35] Wirtz D., Konstantopoulos K., Searson P.C. (2011). The physics of cancer: The role of physical interactions and mechanical forces in metastasis. Nat. Rev. Cancer.

[36] Friedl P., Alexander S. (2011). Cancer invasion and the microenvironment: Plasticity and reciprocity. Cell.

[37] van Helvert S., Storm C., Friedl P. (2018). Mechanoreciprocity in cell migration. Nat. Cell Biol..

[38] Chaudhary P., Yadav K., Lee H.J., Kang K.W., Mo J., Kim J.A. (2024). siRNA treatment targeting integrin α11 overexpressed via EZH2-driven axis inhibits drug-resistant breast cancer progression. Breast Cancer Res..

[39] Zolota V., Tzelepi V., Piperigkou Z., Kourea H., Papakonstantinou E., Argentou Mu I., Karamanos N.K. (2021). Epigenetic Alterations in Triple-Negative Breast Cancer-The Critical Role of Extracellular Matrix. Cancers.

[40] Labani-Motlagh A., Ashja-Mahdavi M., Loskog A. (2020). The Tumor Microenvironment: A Milieu Hindering and Obstructing Antitumor Immune Responses. Front. Immunol..

[41] Wolf K., Te Lindert M., Krause M., Alexander S., Te Riet J., Willis A.L., Hoffman R.M., Figdor C.G., Weiss S.J., Friedl P. (2013). Physical limits of cell migration: Control by ECM space and nuclear deformation and tuning by proteolysis and traction force. J. Cell Biol..

[42] Guzman A., Ziperstein M.J., Kaufman L.J. (2014). The effect of fibrillar matrix architecture on tumor cell invasion of physically challenging environments. Biomaterials.

[43] Qazi T.H., Mooney D.J., Duda G.N., Geissler S. (2017). Biomaterials that promote cell-cell interactions enhance the paracrine function of MSCs. Biomaterials.

[44] Liu Y., Yao X., Zhao Y., Fang D., Shi L., Yang L., Song G., Cai K., Li L., Deng Q. (2023). Mechanotransduction in response to ECM stiffening impairs cGAS immune signaling in tumor cells. Cell Rep..

[45] Zhang M., Zhang B. (2025). Extracellular matrix stiffness: Mechanisms in tumor progression and therapeutic potential in cancer. Exp. Hematol. Oncol..

[46] Feng X., Cao F., Wu X., Xie W., Wang P., Jiang H. (2024). Targeting extracellular matrix stiffness for cancer therapy. Front. Immunol..

[47] Pickup M.W., Mouw J.K., Weaver V.M. (2014). The extracellular matrix modulates the hallmarks of cancer. EMBO Rep..

[48] Henke E., Nandigama R., Ergün S. (2019). Extracellular Matrix in the Tumor Microenvironment and Its Impact on Cancer Therapy. Front. Mol. Biosci..

[49] Laurens N., Koolwijk P., De Maat M.P.M. (2006). Fibrin structure and wound healing. J. Thromb. Haemost..

[50] Foster D.S., Jones R.E., Ransom R.C., Longaker M.T., Norton J.A. (2018). The evolving relationship of wound healing and tumor stroma. JCI Insight.

[51] Wang J.P., Hielscher A. (2017). Fibronectin: How Its Aberrant Expression in Tumors May Improve Therapeutic Targeting. J. Cancer.

[52] Yang J., Song H., Chen L., Cao K., Zhang Y., Li Y., Hao X. (2019). Integrated analysis of microfibrillar-associated proteins reveals MFAP4 as a novel biomarker in human cancers. Epigenomics.

[53] Morra L., Moch H. (2011). Periostin expression and epithelial-mesenchymal transition in cancer: A review and an update. Virchows Arch..

[54] Conway S.J., Izuhara K., Kudo Y., Litvin J., Markwald R., Ouyang G., Arron J.R., Holweg C.T., Kudo A. (2014). The role of periostin in tissue remodeling across health and disease. Cell Mol. Life Sci..

[55] Giblin S.P., Midwood K.S. (2015). Tenascin-C: Form versus function. Cell Adhes. Migr..

[56] Hargadon K.M. (2016). Dysregulation of TGFbeta1 Activity in Cancer and Its Influence on the Quality of Anti-Tumor Immunity. J. Clin. Med..

[57] Xu W., Wang M., Bai Y., Chen Y., Ma X., Yang Z., Zhao L., Li Y. (2022). The role of microfibrillar-associated protein 2 in cancer. Front. Oncol..

[58] Yao L.-W., Wu L.-L., Zhang L.-H., Zhou W., Wu L., He K., Ren J.-C., Deng Y.-C., Yang D.-M., Wang J. (2020). MFAP2 is overexpressed in gastric cancer and promotes motility via the MFAP2/integrin α5β1/FAK/ERK pathway. Oncogenesis.

[59] Gong X., Dong T., Niu M., Liang X., Sun S., Zhang Y., Li Y., Li D. (2020). lncRNA LCPAT1 Upregulation Promotes Breast Cancer Progression via Enhancing MFAP2 Transcription. Mol. Ther. Nucleic Acids.

[60] Gujral T.S., Chan M., Peshkin L., Sorger P.K., Kirschner M.W., MacBeath G. (2014). A noncanonical Frizzled2 pathway regulates epithelial-mesenchymal transition and metastasis. Cell.

[61] Jiang S., Zhang M., Zhang Y., Zhou W., Zhu T., Ruan Q., Chen H., Fang J., Zhou F., Sun J. (2019). WNT5B governs the phenotype of basal-like breast cancer by activating WNT signaling. Cell Commun. Signal..

[62] Xi G., Guo W., Kang D., Ma J., Fu F., Qiu L., Zheng L., He J., Fang N., Chen J. (2021). Large-scale tumor-associated collagen signatures identify high-risk breast cancer patients. Theranostics.

[63] Li Z., Kang D., Xu S., Xi G., Li L., Zheng L., Guo W., Fu F., Wang C., Ma J. (2024). Collagen signature adds prognostically significant information to staging for breast cancer. ESMO Open.

[64] Wang Y., Lu S., Xiong J., Singh K., Hui Y., Zhao C., Brodsky A.S., Yang D., Jolly G., Ouseph M. (2019). ColXα1 is a stromal component that colocalizes with elastin in the breast tumor extracellular matrix. J. Pathol. Clin. Res..

[65] Stewart D.C., Brisson B.K., Dekky B., Berger A.C., Yen W., Mauldin E.A., Loebel C., Gillette D., Assenmacher C.-A., Quincey C. (2024). Prognostic and therapeutic implications of tumor-restrictive type III collagen in the breast cancer microenvironment. Npj Breast Cancer.

[66] Chen P., Cescon M., Bonaldo P. (2013). Collagen VI in cancer and its biological mechanisms. Trends Mol. Med..

[67] Wishart A.L., Conner S.J., Guarin J.R., Fatherree J.P., Peng Y., McGinn R.A., Crews R., Naber S.P., Hunter M., Greenberg A.S. (2020). Decellularized extracellular matrix scaffolds identify full-length collagen VI as a driver of breast cancer cell invasion in obesity and metastasis. Sci. Adv..

[68] Saha S., Duan X., Wu L., Lo P.K., Chen H., Wang Q. (2012). Electrospun fibrous scaffolds promote breast cancer cell alignment and epithelial-mesenchymal transition. Langmuir.

[69] Foroni L., Vasuri F., Valente S., Gualandi C., Focarete M.L., Caprara G., Scandola M., D’Errico-Grigioni A., Pasquinelli G. (2013). The role of 3D microenvironmental organization in MCF-7 epithelial-mesenchymal transition after 7 culture days. Exp. Cell Res..

[70] Tien J., Ghani U., Dance Y.W., Seibel A.J., Karakan M., Ekinci K.L., Nelson C.M. (2020). Matrix Pore Size Governs Escape of Human Breast Cancer Cells from a Microtumor to an Empty Cavity. iScience.

[71] Saif Ur Rahman M., Wu J., Chen H., Sun C., Liu Y., Xu S. (2023). Matrix mechanophysical factor: Pore size governs the cell behavior in cancer. Adv. Phys. X.

