# Supplementary material for: Evaluation of Metaplastic Triple-Negative Breast Cancer Extracellular Matrix Structure and Protein Composition
Source: Bioengineering (Basel). 2025 Dec 31;13(1):47. doi: 10.3390/bioengineering13010047 (PMC12838215; doi:10.3390/bioengineering13010047)
Supplement: Supplementary file 1 [file bioengineering-13-00047-s001.zip › bioengineering-4003838-supplementary.pdf]

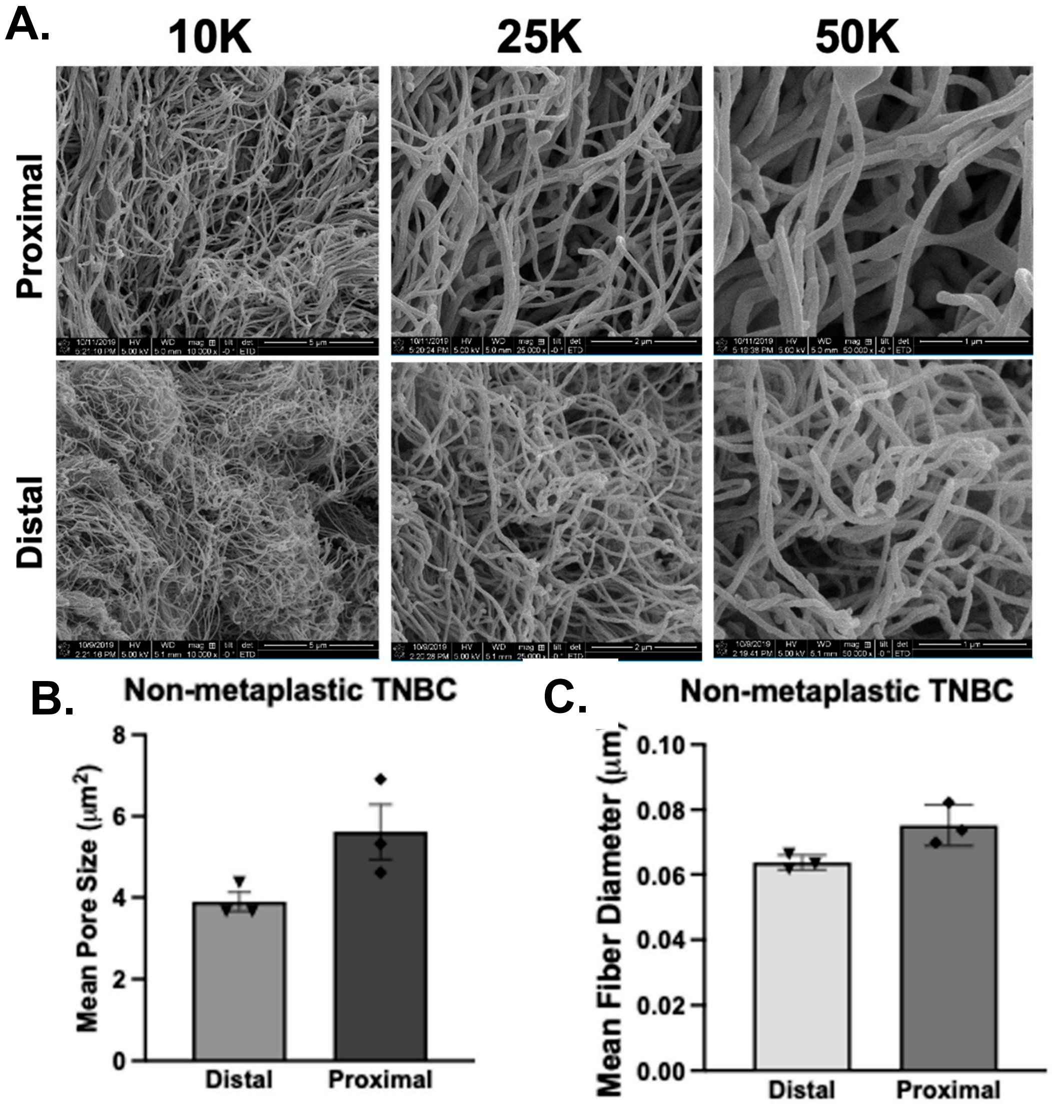

Supplemental Figure S1: SEM analysis was completed on distal and proximal non-metaplastic TNBC tissues at (A) 10k, 25k, and 50k magnification, where (B) pore size and (C) mean fiber thickness (diameter) were calculated in 3 independent experiments ± SEM.

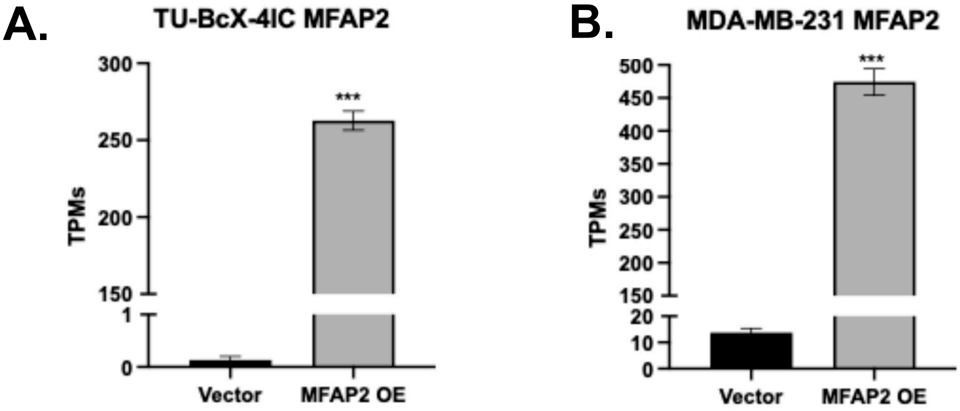

Supplemental Figure S2: RNA sequencing was performed on TU-BcX-4IC and MDA-MB-231 cell lines with induced expression of MFAP2 or vector control. Transcripts per million (TPMs) confirmation MFAP2 overexpression in (A) Tu-BcX-4IC and (B) MDA-MB-231 cells after transfection. N=3 biological replicates from pooled cell lines. *** significantly different p<0.001. Error bars represent SEM.

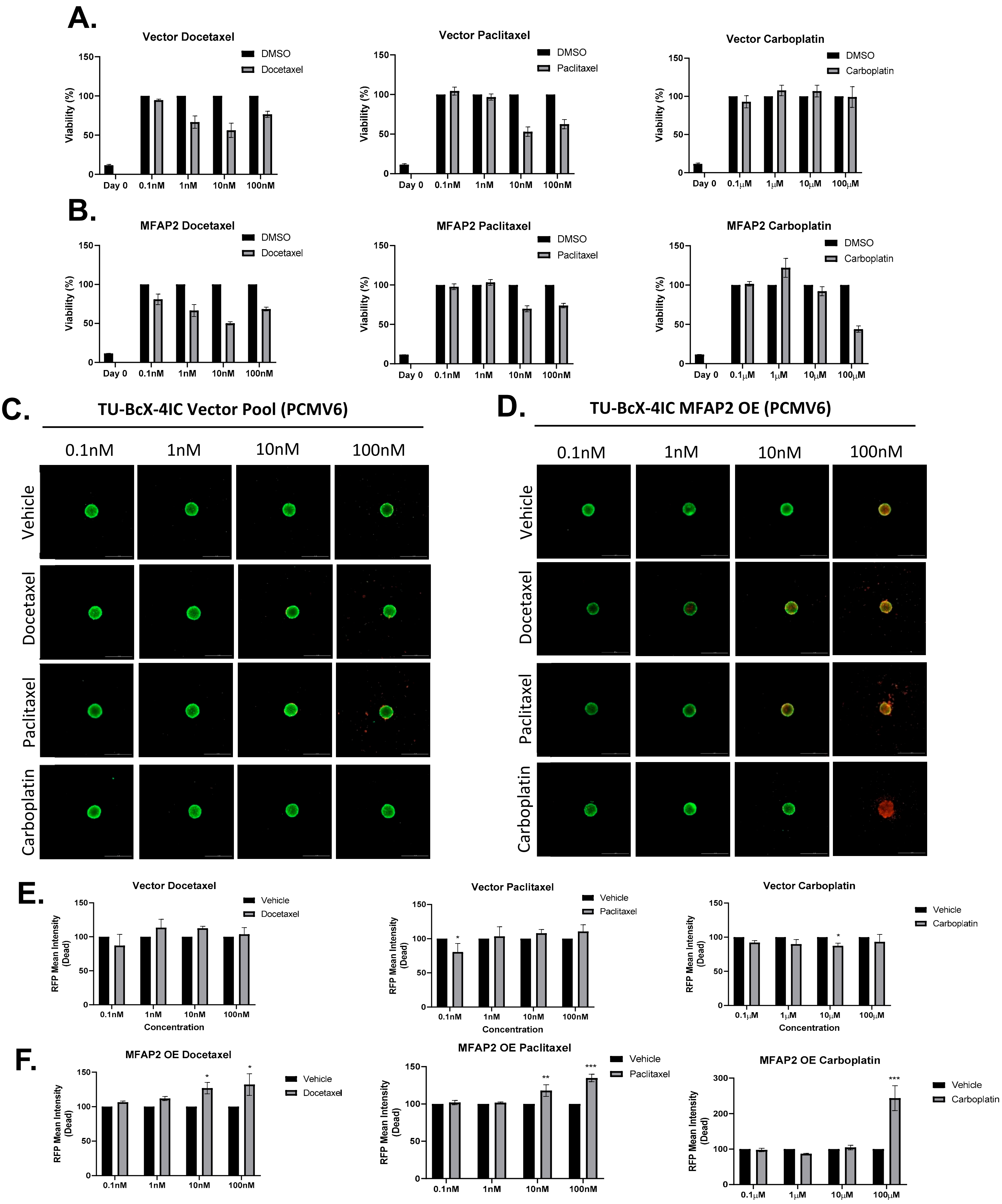
A.
Vector Docetaxel
DMSO
Docetaxel
Viability (%)
Day 0
0.1nM
1nM
10nM
100nM
Vector Paclitaxel
Paclitaxel
Vector Carboplatin
Carboplatin
0.1μM
1μM
10μM
100μM
B.
MFAP2 Docetaxel
MFAP2 Paclitaxel
MFAP2 Carboplatin
C.
TU-BcX-4IC Vector Pool (PCMV6)
D.
TU-BcX-4IC MFAP2 OE (PCMV6)
Vehicle
Docetaxel
Paclitaxel
Carboplatin
E.
Vector Docetaxel
Vector Paclitaxel
Vector Carboplatin
RFP Mean Intensity (Dead)
Concentration
F.
MFAP2 OE Docetaxel
MFAP2 OE Paclitaxel
MFAP2 OE Carboplatin

Supplemental Figure S3: (A) A crystal violet assay was used to determine a therapeutic response for Tu-BcX-4IC pCMV6 Vector and (B) MFAP2 OE cells treated with docetaxel, paclitaxel, and carboplatin for 72 hrs in 2D culture. Percent average viability for the chemotherapy treatments was normalized to vehicle-treated wells in triplicate experiments. (C) Tu-BcX-4IC pCMV6 Vector spheroids and (D) MFAP2 OE spheroids were treated at various dosages of docetaxel, paclitaxel, and carboplatin for 72 hrs and then stained with Live/Dead stain to determine a dose curve. Representative images show merged RFP and GFP channels after 72 hrs of treatment. (E) The RFP mean intensity, representing a fluorescent signal from the dead Tu-BcX-4IC Vector cells, and (F) MFAP2 OE cells, were graphed as a precent average ± SEM in n= biological triplicate experiments. $^{*}p \leq 0.05$; $^{**}p \leq 0.01$; $^{***}p \leq 0.001$.

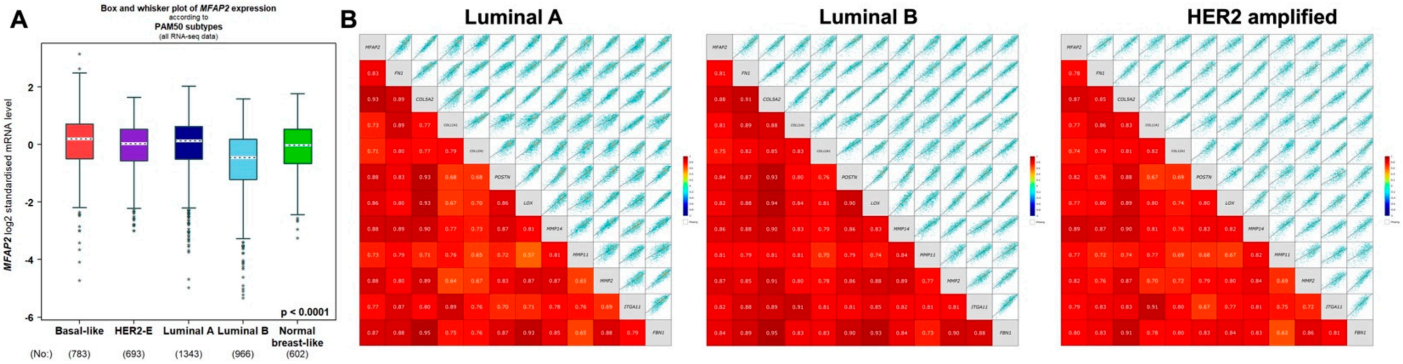

Supplemental Figure S4: MFAP2 expression and correlation mapping across the breast cancer subtypes. (A) Box and whisker blot of MFAP2 expression across breast cancer subtypes (PAM50 classification). Significance determined by Welch's test. Expression profiles obtained from the BC-GenExMiner (v5.2). n= 4421 patients were evaluated from three RNA-seq datasets: TCGA, SCAN-B GSE96058, and SCAN-B GSE81538. Access date December 2025. (B) Correlation map for gene expression of MFAP2-associated genes specific to EMT/invasion and matrix remodeling. Pathway and correlation maps were generated form publicly available RNA sequencing data with the BC-GenExMiner (v5.2) for the breast cancer molecular subtypes Luminal A, Luminal B, and HER2 amplified. Access date December 2025.
