# Supplementary material for: Evaluation of Metaplastic Triple-Negative Breast Cancer Extracellular Matrix Structure and Protein Composition
Source: Bioengineering (Basel). 2025 Dec 31;13(1):47. doi: 10.3390/bioengineering13010047 (PMC12838215; doi:10.3390/bioengineering13010047)
Supplement: Supplementary file 1 [file bioengineering-13-00047-s001.zip › Supplemental Table S1.pdf]

	Distal		Proximal		Tumor	
**Angle**	Normalized Frequency	Orientation Index	Normalized Frequency	Orientation Index	Normalized Frequency	Orientation Index
**-90**	0.00754	0.00529	0.00118	0.00083	0.01114	0.00780
**-89**	0.00727	0.00553	0.00109	0.00083	0.01267	0.00963
**-88**	0.00720	0.01079	0.00070	0.00104	0.01135	0.01701
**-87**	0.00687	0.00566	0.00034	0.00028	0.01128	0.00930
**-86**	0.00673	0.00436	0.00029	0.00019	0.00990	0.00641
**-85**	0.00730	0.01072	0.00027	0.00039	0.00875	0.01285
**-84**	0.00732	0.00704	0.00041	0.00039	0.00852	0.00820
**-83**	0.00753	0.00423	0.00061	0.00034	0.00860	0.00484
**-82**	0.00840	0.01178	0.00058	0.00081	0.00823	0.01154
**-81**	0.00836	0.00923	0.00046	0.00051	0.00795	0.00877
**-80**	0.00737	0.00377	0.00052	0.00027	0.00746	0.00382
**-79**	0.00651	0.00848	0.00078	0.00101	0.00741	0.00965
**-78**	0.00629	0.00778	0.00063	0.00078	0.00723	0.00894
**-77**	0.00662	0.00332	0.00050	0.00025	0.00719	0.00360
**-76**	0.00680	0.00802	0.00023	0.00027	0.00768	0.00906
**-75**	0.00669	0.00903	0.00003	0.00005	0.00819	0.01105
**-74**	0.00606	0.00321	0.00000	0.00000	0.00839	0.00444
**-73**	0.00585	0.00609	0.00003	0.00004	0.00774	0.00806
**-72**	0.00626	0.00898	0.00006	0.00009	0.00757	0.01086
**-71**	0.00624	0.00371	0.00017	0.00010	0.00758	0.00451
**-70**	0.00594	0.00535	0.00027	0.00024	0.00737	0.00664
**-69**	0.00605	0.00900	0.00015	0.00023	0.00750	0.01116
**-68**	0.00666	0.00462	0.00027	0.00019	0.00811	0.00563
**-67**	0.00766	0.00588	0.00045	0.00034	0.00908	0.00697
**-66**	0.00650	0.00975	0.00070	0.00106	0.01000	0.01499
**-65**	0.00561	0.00458	0.00081	0.00066	0.01045	0.00853
**-64**	0.00512	0.00334	0.00107	0.00070	0.01163	0.00760
**-63**	0.00442	0.00650	0.00137	0.00201	0.01276	0.01879
**-62**	0.00468	0.00446	0.00196	0.00187	0.01368	0.01304
**-61**	0.00615	0.00348	0.00284	0.00161	0.01371	0.00777
**-60**	0.00594	0.00836	0.00378	0.00532	0.01280	0.01801
**-59**	0.00578	0.00633	0.00420	0.00460	0.01262	0.01381
**-58**	0.00459	0.00236	0.00509	0.00262	0.01269	0.00652
**-57**	0.00369	0.00483	0.00615	0.00805	0.01286	0.01685
**-56**	0.00326	0.00400	0.00782	0.00960	0.01338	0.01643
**-55**	0.00297	0.00149	0.00839	0.00420	0.01392	0.00697
**-54**	0.00273	0.00325	0.00933	0.01108	0.01393	0.01655
**-53**	0.00361	0.00485	0.01147	0.01540	0.01490	0.02002
**-52**	0.00292	0.00154	0.01276	0.00672	0.01633	0.00860
**-51**	0.00256	0.00269	0.01401	0.01472	0.01706	0.01793
**-50**	0.00292	0.00417	0.01512	0.02164	0.01768	0.02530
**-49**	0.00323	0.00191	0.01677	0.00990	0.01792	0.01058
**-48**	0.00337	0.00306	0.01630	0.01483	0.01749	0.01591
**-47**	0.00337	0.00500	0.01398	0.02076	0.01814	0.02693
**-46**	0.00343	0.00235	0.01334	0.00916	0.01928	0.01324
**-45**	0.00354	0.00275	0.01254	0.00973	0.01964	0.01524
**-44**	0.00346	0.00519	0.01203	0.01805	0.02072	0.03107
**-43**	0.00354	0.00286	0.01197	0.00968	0.02118	0.01711
**-42**	0.00319	0.00211	0.01120	0.00739	0.02004	0.01323
**-41**	0.00324	0.00477	0.01120	0.01652	0.01773	0.02614
**-40**	0.00286	0.00270	0.01097	0.01037	0.01510	0.01426
**-39**	0.00304	0.00173	0.01134	0.00648	0.01267	0.00724
**-38**	0.00251	0.00355	0.01115	0.01574	0.01153	0.01628

**-37**	0.00173	0.00188	0.01051	0.01141	0.01073	0.01165
**-36**	0.00126	0.00065	0.01065	0.00550	0.01022	0.00527
**-35**	0.00106	0.00140	0.01093	0.01439	0.00955	0.01258
**-34**	0.00081	0.00099	0.01139	0.01390	0.00862	0.01052
**-33**	0.00048	0.00024	0.01080	0.00540	0.00792	0.00396
**-32**	0.00014	0.00017	0.00989	0.01183	0.00790	0.00944
**-31**	0.00004	0.00005	0.00928	0.01241	0.00806	0.01078
**-30**	0.00005	0.00003	0.01059	0.00555	0.00768	0.00402
**-29**	0.00022	0.00023	0.01051	0.01113	0.00731	0.00774
**-28**	0.00019	0.00027	0.01012	0.01444	0.00733	0.01045
**-27**	0.00000	0.00000	0.00998	0.00584	0.00744	0.00435
**-26**	0.00019	0.00018	0.00963	0.00885	0.00806	0.00740
**-25**	0.00062	0.00092	0.00951	0.01409	0.00778	0.01153
**-24**	0.00034	0.00023	0.00943	0.00641	0.00788	0.00536
**-23**	0.00017	0.00014	0.00942	0.00738	0.00751	0.00589
**-22**	0.00005	0.00007	0.00913	0.01370	0.00698	0.01047
**-21**	0.00006	0.00005	0.00889	0.00711	0.00661	0.00529
**-20**	0.00007	0.00005	0.00882	0.00588	0.00646	0.00431
**-19**	0.00023	0.00034	0.00913	0.01348	0.00609	0.00900
**-18**	0.00077	0.00072	0.00929	0.00869	0.00587	0.00549
**-17**	0.00173	0.00100	0.00957	0.00551	0.00582	0.00335
**-16**	0.00287	0.00407	0.00907	0.01285	0.00558	0.00791
**-15**	0.00317	0.00341	0.00895	0.00963	0.00520	0.00560
**-14**	0.00243	0.00126	0.00891	0.00462	0.00489	0.00254
**-13**	0.00272	0.00361	0.00915	0.01211	0.00466	0.00617
**-12**	0.00297	0.00360	0.01002	0.01215	0.00453	0.00549
**-11**	0.00278	0.00139	0.01063	0.00531	0.00429	0.00214
**-10**	0.00252	0.00303	0.01052	0.01266	0.00419	0.00504
**-9**	0.00261	0.00347	0.01032	0.01372	0.00417	0.00554
**-8**	0.00319	0.00166	0.01092	0.00569	0.00402	0.00209
**-7**	0.00338	0.00361	0.01197	0.01279	0.00400	0.00427
**-6**	0.00381	0.00541	0.01240	0.01763	0.00412	0.00585
**-5**	0.00576	0.00334	0.01245	0.00723	0.00466	0.00270
**-4**	0.00670	0.00621	0.00987	0.00915	0.00341	0.00317
**-3**	0.00612	0.00906	0.00939	0.01389	0.00240	0.00355
**-2**	0.00524	0.00353	0.01006	0.00677	0.00242	0.00163
**-1**	0.00392	0.00311	0.01108	0.00877	0.00237	0.00188
**0**	0.00297	0.00446	0.00933	0.01399	0.00315	0.00472
**1**	0.00257	0.00204	0.00875	0.00693	0.00207	0.00164
**2**	0.00243	0.00164	0.00862	0.00580	0.00124	0.00083
**3**	0.00279	0.00412	0.00740	0.01095	0.00117	0.00172
**4**	0.00288	0.00267	0.00718	0.00666	0.00083	0.00077
**5**	0.00310	0.00180	0.00897	0.00521	0.00053	0.00031
**6**	0.00339	0.00482	0.00831	0.01182	0.00040	0.00056
**7**	0.00310	0.00331	0.00762	0.00815	0.00038	0.00040
**8**	0.00325	0.00169	0.00778	0.00405	0.00042	0.00022
**9**	0.00341	0.00453	0.00872	0.01159	0.00052	0.00069
**10**	0.00400	0.00482	0.00838	0.01009	0.00044	0.00053
**11**	0.00416	0.00208	0.00793	0.00397	0.00040	0.00020
**12**	0.00482	0.00584	0.00757	0.00917	0.00034	0.00042
**13**	0.00725	0.00959	0.00760	0.01006	0.00058	0.00076
**14**	0.00611	0.00317	0.00779	0.00404	0.00076	0.00040
**15**	0.00333	0.00359	0.00812	0.00874	0.00079	0.00086
**16**	0.00266	0.00377	0.00796	0.01128	0.00078	0.00111
**17**	0.00263	0.00152	0.00795	0.00458	0.00080	0.00046
**18**	0.00246	0.00230	0.00635	0.00594	0.00097	0.00090

**19**	0.00244	0.00361	0.00592	0.00874	0.00091	0.00135
**20**	0.00220	0.00146	0.00591	0.00394	0.00071	0.00047
**21**	0.00260	0.00208	0.00553	0.00442	0.00062	0.00049
**22**	0.00282	0.00423	0.00535	0.00803	0.00066	0.00099
**23**	0.00309	0.00242	0.00542	0.00425	0.00076	0.00060
**24**	0.00295	0.00201	0.00569	0.00387	0.00094	0.00064
**25**	0.00314	0.00465	0.00548	0.00812	0.00085	0.00125
**26**	0.00373	0.00342	0.00518	0.00476	0.00061	0.00056
**27**	0.00374	0.00219	0.00540	0.00316	0.00045	0.00026
**28**	0.00409	0.00583	0.00537	0.00766	0.00050	0.00072
**29**	0.00353	0.00374	0.00514	0.00544	0.00060	0.00064
**30**	0.00325	0.00170	0.00524	0.00274	0.00074	0.00039
**31**	0.00289	0.00386	0.00550	0.00735	0.00091	0.00121
**32**	0.00283	0.00338	0.00540	0.00645	0.00113	0.00135
**33**	0.00332	0.00166	0.00588	0.00294	0.00149	0.00075
**34**	0.00430	0.00525	0.00614	0.00749	0.00118	0.00144
**35**	0.00490	0.00645	0.00525	0.00691	0.00092	0.00122
**36**	0.00563	0.00291	0.00548	0.00283	0.00085	0.00044
**37**	0.00595	0.00646	0.00571	0.00620	0.00098	0.00107
**38**	0.00554	0.00782	0.00587	0.00829	0.00080	0.00112
**39**	0.00540	0.00308	0.00589	0.00336	0.00077	0.00044
**40**	0.00562	0.00531	0.00516	0.00488	0.00079	0.00074
**41**	0.00581	0.00857	0.00514	0.00758	0.00083	0.00123
**42**	0.00619	0.00409	0.00550	0.00363	0.00111	0.00073
**43**	0.00648	0.00524	0.00566	0.00457	0.00169	0.00137
**44**	0.00679	0.01018	0.00511	0.00767	0.00087	0.00131
**45**	0.00727	0.00564	0.00499	0.00387	0.00015	0.00012
**46**	0.00807	0.00554	0.00448	0.00308	0.00000	0.00000
**47**	0.00824	0.01223	0.00399	0.00593	0.00001	0.00002
**48**	0.00803	0.00731	0.00330	0.00300	0.00030	0.00027
**49**	0.00840	0.00496	0.00297	0.00175	0.00056	0.00033
**50**	0.00903	0.01293	0.00351	0.00502	0.00062	0.00089
**51**	0.00989	0.01040	0.00358	0.00376	0.00060	0.00064
**52**	0.01168	0.00615	0.00273	0.00144	0.00076	0.00040
**53**	0.01354	0.01819	0.00234	0.00314	0.00110	0.00148
**54**	0.01366	0.01623	0.00191	0.00227	0.00093	0.00110
**55**	0.01331	0.00666	0.00155	0.00078	0.00088	0.00044
**56**	0.01268	0.01557	0.00154	0.00189	0.00061	0.00075
**57**	0.01141	0.01495	0.00168	0.00221	0.00036	0.00048
**58**	0.01140	0.00586	0.00166	0.00085	0.00018	0.00009
**59**	0.01365	0.01494	0.00162	0.00177	0.00008	0.00008
**60**	0.01416	0.01993	0.00165	0.00233	0.00003	0.00004
**61**	0.01312	0.00743	0.00181	0.00103	0.00001	0.00001
**62**	0.01437	0.01371	0.00189	0.00180	0.00006	0.00005
**63**	0.01478	0.02175	0.00150	0.00220	0.00011	0.00016
**64**	0.01365	0.00892	0.00122	0.00080	0.00015	0.00010
**65**	0.01364	0.01114	0.00102	0.00083	0.00044	0.00036
**66**	0.01327	0.01989	0.00079	0.00119	0.00051	0.00076
**67**	0.01237	0.00950	0.00048	0.00037	0.00044	0.00034
**68**	0.01131	0.00785	0.00016	0.00011	0.00049	0.00034
**69**	0.01151	0.01711	0.00012	0.00018	0.00062	0.00093
**70**	0.01139	0.01026	0.00027	0.00025	0.00089	0.00080
**71**	0.01110	0.00661	0.00027	0.00016	0.00111	0.00066
**72**	0.01099	0.01578	0.00028	0.00040	0.00131	0.00188
**73**	0.01086	0.01131	0.00040	0.00041	0.00151	0.00157
**74**	0.01105	0.00585	0.00057	0.00030	0.00170	0.00090

**75**	0.01226	0.01655	0.00063	0.00085	0.00187	0.00253
**76**	0.01279	0.01509	0.00072	0.00085	0.00231	0.00273
**77**	0.01004	0.00503	0.00068	0.00034	0.00287	0.00144
**78**	0.00874	0.01080	0.00044	0.00055	0.00367	0.00453
**79**	0.00773	0.01007	0.00022	0.00029	0.00474	0.00618
**80**	0.00723	0.00370	0.00012	0.00006	0.00488	0.00250
**81**	0.00753	0.00831	0.00014	0.00016	0.00478	0.00527
**82**	0.00734	0.01029	0.00033	0.00046	0.00449	0.00630
**83**	0.00646	0.00363	0.00031	0.00017	0.00376	0.00211
**84**	0.00714	0.00687	0.00033	0.00032	0.00382	0.00368
**85**	0.00780	0.01146	0.00050	0.00073	0.00449	0.00660
**86**	0.00822	0.00532	0.00060	0.00039	0.00591	0.00383
**87**	0.00832	0.00686	0.00059	0.00049	0.00578	0.00477
**88**	0.00815	0.01222	0.00071	0.00106	0.00663	0.00993
